# Direct Identification of O─O Bond Formation Through Three‐Step Oxidation During Water Splitting by Operando Soft X‐ray Absorption Spectroscopy

**DOI:** 10.1002/advs.202401236

**Published:** 2024-08-01

**Authors:** Yu‐Cheng Huang, Yujie Wu, Ying‐Rui Lu, Jeng‐Lung Chen, Hong‐Ji Lin, Chien‐Te Chen, Chi‐Liang Chen, Chao Jing, Jing Zhou, Linjuan Zhang, Yanyong Wang, Wu‐Ching Chou, Shuangyin Wang, Zhiwei Hu, Chung‐Li Dong

**Affiliations:** ^1^ National Synchrotron Radiation Research Center Hsinchu 30076 Taiwan; ^2^ Department of Electrophysics National Yang Ming Chiao Tung University Hsinchu 300093 Taiwan; ^3^ State Key Laboratory of Chemo/Bio‐Sensing and Chemometrics College of Chemistry and Chemical Engineering Advanced Catalytic Engineering Research Center of the Ministry of Education Hunan University Changsha 410082 China; ^4^ Key Laboratory of Interfacial Physics and Technology Shanghai Institute of Applied Physics Chinese Academy of Sciences Shanghai 201800 China; ^5^ Max‐Planck‐Institute for Chemical Physics of Solids 01187 Dresden Germany; ^6^ Research Center for X‐ray Science & Department of Physics Tamkang University New Taipei 25137 Taiwan

**Keywords:** conjugated chromium oxalate Anions [Cr(C_2_O_4_)_3_]^3−^, Layered Double Hydroxides, Operando soft X‐ray Spectroscopy, Oxygen Evolution Reaction, Oxyhydroxide

## Abstract

Anionic redox allows the direct formation of O─O bonds from lattice oxygens and provides higher catalytic in the oxygen evolution reaction (OER) than does the conventional metal ion mechanism. While previous theories have predicted and experiments have suggested the possible O─O bond, it has not yet been directly observed in the OER process. In this study, operando soft X‐ray absorption spectroscopy (sXAS) at the O K‐edge and the operando Raman spectra is performed on layered double CoFe hydroxides (LDHs) after intercalation with [Cr(C_2_O_4_)_3_]^3−^, and revealed a three‐step oxidation process, staring from Co^2+^ to Co^3+^, further to Co^4+^ (3d6L), and ultimately leading to the formation of O─O bonds and O_2_ evolution above a threshold voltage (1.4 V). In contrast, a gradual oxidation of Fe is observed in CoFe LDHs. The OER activity exhibits a significant enhancement, with the overpotential decreasing from 300 to 248 mV at 10 mA cm^−2^, following the intercalation of [Cr(C_2_O_4_)_3_]^3−^ into CoFe LDHs, underscoring a crucial role of anionic redox in facilitating water splitting.

## Graphical abstract

This study reports the first direct observation of O─O bond formation during the OER using operando soft XAS and Raman spectroscopy on layered double hydroxides intercalated with [Cr(C_2_O_4_)_3_]^3−^. This work reveals a stepwise oxidation process leading to the formation of O─O bonds and O_2_ evolution, highlighting the crucial role of anionic redox in facilitating water splitting.

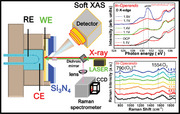

## 1 Introduction

The environmentally friendly electrochemical oxygen evolution reaction (OER) for splitting water provides high‐purity hydrogen and oxygen with abundant resources, and so it is a means of so‐called “green hydrogen” production. However, the OER is also the rate‐limiting step in water splitting due to its slow kinetics. Thus, understanding the origin of OER activity and identifying the real active species are very important to the development of efficient and low‐cost OER electrocatalysts with high activity and durability for industrial use.^[^ ^1^, ^2^, ^3^, ^4^ ^]^ Although, in the past decades, numerous electrocatalysts have been investigated with a view to reducing the overpotential of the OER in order to improve its energy conversion efficiency, noble metal oxides OER catalysts, such as IrO_2_ and RuO_2_, are still considered state‐of‐the‐art OER catalysts.^[^ ^5^, ^6^ ^]^ However, these materials are costly, scarce, and relatively unstable, hindering their large‐scale use in industry.^[^ ^7^, ^8^ ^]^ Therefore, the development of earth‐abundant catalysts for the OER that are highly efficient and stable is highly desirable.

Among various noble‐metal‐free electrocatalyst candidates, 3d transition metal oxides and hydroxides are a class of materials that perform well in the OER.^[^ ^1^, ^2^, ^4^, ^9^ ^]^ Recently, brucite‐like structures of transition metal oxides, called layered double hydroxides (LDHs), have been discovered, and they exhibit very interesting and remarkable properties in OER^[^ ^10^, ^11^, ^12^, ^13^ ^]^ LDHs are abundant, low‐cost, easily synthesized, and compositionally tunable; they also have numerous physiochemical degrees of freedom and so are brought to the forefront of OER research. Among the LDHs catalysts, NiFe LDHs have attracted much attention owing to their high activity and stability in alkaline solutions. Expanding the interlayer distance, and even exfoliation, can create more active sites and improve electrocatalytic activity, improving their otherwise poor conductivity. For example, Chen et al. reported that changing the interlayer spacing from 2.9 to 5.8 Å by introducing PtCl62‐ to replace CO_3_^2−^ improved the catalytic activity of NiFe LDHs in the OER.^[^ ^14^ ^]^ The large anionic oxalate complexes [Cr(C_2_O_4_)_3_]^3−^ not only increase the substrate spacing of LDHs, but also effectively tune their electronic structures.^[^ ^2^, ^15^, ^16^, ^17^ ^]^ Such metallic complexes can maintain the structural stability of LDHs by hydrogen‐bonding with host‐layer hydroxyl groups.

Whereas Ni‐based LDHs have been widely investigated, only a few Co‐based LDHs have been reported upon,^[^ ^18^, ^19^, ^20^ ^]^ despite the well‐known high activity of Co‐based oxides for the OER. Additionally, the incorporation of Fe greatly enhances their OER activity over that of pure Co‐based catalysts. Anionic redox, specifically the role of lattice oxygen and an active oxygen species, has garnered significant attention and is frequently discussed in the field of electrochemical catalysis recently. The well‐known active oxygen species in the OER is 3dnL (where the L stands for an O 2p hole and n = 6/7 for Co^3+^/2+ as‐prepared materials).^[^ ^21^, ^22^, ^23^ ^]^ Some reports have suggested the formation of µ‐OO peroxide (Co‐OO‐Co) moieties and superoxo/peroxo‐like (O_2_)n− species using in situ/operando Raman spectroscopy.^[^ ^24^, ^25^, ^26^ ^]^ The onset of the catalytic OER has been proposed to be intimately coupled to the formal Co^3+^/Co^4+^ redox transition, leading to protonatable µ2‐O sites as well as oxidized Co+3+δ ions that are bound to terminal O‐(2‐δ) ligands.^[^ ^4^ ^]^ Notably, no clearly O─O bond‐related spectral features that are predicted by theory have yet been observed by O K‐edge XAS under OER conditions. Nevertheless, the formation of O2‐ species at a photon energy above 531 eV was recently observed in the O K‐edge XAS spectrum of Na0.6[Li0.2Mn0.8]O_2_ batteries.^[^ ^27^ ^]^

In recent years, operando hard X‐ray absorption near‐edge structures (XANES) and extended X‐ray absorption fineness (EXAFS) on the K‐edges of 3d/4d transition elements, and the L2,3‐edges of 5d elements have become standard spectroscopic techniques for characterizing the electronic structures and local atomic environments owing to their ease of implementation under atmospheric conditions.^[^ ^14^, ^28^, ^29^ ^]^ The hard X‐ray absorption spectra (hXAS) at the 3d K‐edge have been successfully employed to explore the formation of high oxidation states (Fe^4+^, Co^4+^, Ni^4+^, Cu^3+^)^[^ ^7^, ^29^, ^30^, ^31^, ^32^ ^]^ and the structural modifications in the respective 3d elemental oxides that are formed under the operando condition,^[^ ^33^, ^34^ ^]^ but they cannot be used to distinguish µ‐OH species from the ligand hole state 3dn+1L in high oxidation states, as the holes that are created during the OER are primarily located in O 2p orbitals.^[^ ^35^, ^36^ ^]^ The soft X‐ray absorption spectroscopy (sXAS) at the O K‐edge is the most direct and effective experimental tool for studying the possible formation of ligand holes from O 2p orbitals.^[^ ^24^, ^37^, ^38^, ^39^ ^]^ Therefore, O K‐edge sXAS spectroscopy is often used to determine Co^4+^ content because of Co3d‐O2p covalent mixing.^[^ ^21^, ^31^, ^35^, ^40^, ^41^ ^]^ The ex situ sXAS experiments might be capable of identifying 3dn+1L and (O_2_)n−, if the reaction being studied is irreversible and these states under certain OER persist even after removing the voltage.^[^ ^35^ ^]^ The reversible OER reaction requires an operando O K‐edge sXAS spectroscopic study, but the harsh experimental condition must be overcome by separating an electrochemical liquid cell from an ultra‐high vacuum in the soft X‐ray region.

Herein, the rational regulation of the active Co center with unsaturated coordination (CoFe‐[Cr(C_2_O_4_)_3_]^3−^‐LDHs) via the intercalation of conjugated chromium oxalate anions [Cr(C_2_O_4_)_3_]^3−^ is systematically studied by a combination of in situ sXAS, hXAS, XRD and Raman spectroscopy. We observe the (O_2_)n‐related spectral feature in addition to the Co4+ intermediate, for the first time in the O K‐edge XAS spectra under strongly reactive OER conditions in CoFe‐[Cr(C_2_O_4_)_3_]^3−^‐LDHs, but not in pure CoFe‐LDHs. The in situ Raman spectra further indicate that Co(oxyhydr)oxides and O_2_ are formed simultaneously.

## 2 Results and Discussion

### 2.1 Catalyst Characterization

**Figure 1:**
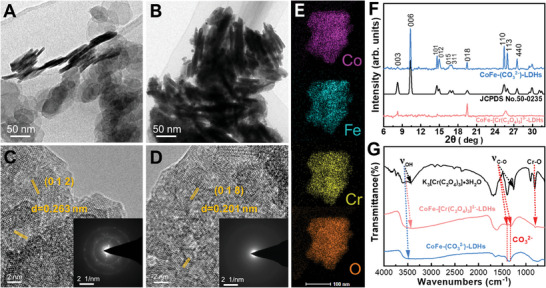
A) TEM image of CoFe‐(CO_3_^2−^)‐LDHs. B) TEM image of CoFe‐[Cr(C_2_O_4_)_3_]^3−^‐LDHs; C) HRTEM image of CoFe‐(CO_3_^2−^)‐LDHs and corresponding SAED (inset). D) HRTEM image of CoFe‐[Cr(C_2_O_4_)_3_]^3−^‐LDHs and corresponding SAED (inset). E) EDX mapping images of CoFe‐[Cr(C_2_O_4_)_3_]^3−^‐LDHs. F) XRD patterns of as‐prepared CoFe‐(CO_3_^2−^)‐LDHs and CoFe‐[Cr(C_2_O_4_)_3_]^3−^‐LDHs. G) FTIR spectra of as‐synthesized K3[Cr(C_2_O_4_)3]·3H2O, CoFe‐(CO_3_^2−^)‐LDHs, and CoFe‐[Cr(C_2_O_4_)_3_]^3−^‐LDHs.

The morphology and microstructure of the obtained samples were observed by transmission electron microscopy (TEM) and scanning electron microscopy (SEM). Typically, CoFe‐(CO_3_^2−^)‐LDHs exhibit sheet‐like morphology with a lateral size of ≈10–40 nm in a hexagonal shape (**Figure** **1**A; Figure S1A Supporting Information). After the intercalation of [Cr(C_2_O_4_)_3_]^3−^, CoFe‐[Cr(C_2_O_4_)_3_]^3−^‐LDHs maintain their brucite‐like structure (Figure 1B; Figure S1B Supporting Information). According to high‐resolution TEM (HRTEM) analysis, the lattice fringe spacing of CoFe‐(CO_3_^2−^)‐LDHs and CoFe‐[Cr(C_2_O_4_)_3_]^3−^‐LDHs is 0.263 and 0.201 nm, respectively (Figure 1C,D), corresponding to (012) and (018) planes, which are characteristic of CoFe‐(CO_3_^2−^)‐LDHs (JPCDS. No 50–0235), indicating the preservation of the crystal structure was maintained following intercalation. Notably, exposing the high‐index crystal face (018) with high surface energy often leads to more efficient catalytic activity due to the increased surface chemical reactivity.^[^ ^42^ ^]^

The energy‐dispersive X‐ray (EDX) spectroscopy mapping images confirm the uniform distribution of elements Co, Fe, Cr, and O in CoFe‐[Cr(C_2_O_4_)_3_]^3−^‐LDHs (Figure 1E), further revealing the presence of [Cr(C_2_O_4_)_3_]^3−^‐ intercalation anions. Figure 1F shows the XRD patterns of the as‐prepared CoFe‐(CO_3_^2−^)‐LDHs and CoFe‐[Cr(C_2_O_4_)_3_]^3−^‐LDHs powders, which exhibit the typical characteristics of LDHs with a brucite‐like structure with a hexagonal unit cell of LDHs.^[^ ^43^ ^]^ The observed diffraction peaks at 8.2°, 10.35°, 14.6°, 15°, 16.7°, 17.1°, 19.5°, 25.5° and 25.95° are indexed to the (003), (006), (101), (012), (015), (311), (018), (110), and (113) planes of the CoFe‐(CO32‐)‐LDHs structure (JPCDS. No 50–0235), respectively. The (006) basal plane spacing is determined to be 10.35°, which is consistent with that of CoFe LDH reported previously.^[^ ^44^ ^]^ The (006) basal plane spacing suggests that only CO32‐ acts as the charge‐compensating anion in the CoFe‐(CO_3_^2−^)‐LDHs, because the CO_3_^2−^ in the precursor solution has a strong affinity for the positively charged brucite‐like layer.^[^ ^45^ ^]^

The larger size of [Cr(C_2_O_4_)_3_]^3−^ intercalation significantly affects the crystallinity of CoFe LDHs, leading to the decrease in crystallinity in CoFe‐[Cr(C_2_O_4_)_3_]^3−^LDHs, as indicated by a single plane observed at 19.5°, highlighting the influence of heavy interlayer molecules like chromate in CoFe LDHs, which results in an increased interlayer distance. To further identify the anions located in the LDHs, Fourier‐transform infrared spectroscopy (FTIR) was conducted to analyze the CoFe LDHs, and the spectrum between 400 and 4000 cm^−1^ is shown in Figure 1G. The band located at 813 cm^−1^ is identified as the stretching vibration of Cr‐O in K_3_[Cr(C_2_O_4_)^3^]·3H_2_O and CoFe‐[Cr(C_2_O_4_)_3_]^3−^‐LDHs. K_3_[Cr(C_2_O_4_)^3^]·3H_2_O yields two peaks at 1390 and 1265 cm^−1^, which are assigned to CO symmetric vibration modes of oxalate.^[^ ^16^ ^]^ For CoFe‐[Cr(C_2_O_4_)_3_]^3−^‐LDHs, the peak position of the CO symmetric vibration changes slightly with the interaction between intercalated [Cr(C_2_O_4_)_3_]^3−^ and the hydroxide layer. The broad band at 3409 cm^−1^ is ascribed to the O─H stretching vibration mode of water and hydroxyl groups in the LDH; the shoulder at 3050 cm^−1^ is attributed to hydroxyl interactions with carbonate ions in the interlayer.^[^ ^46^ ^]^ All of these results confirm the intercalation of [Cr(C_2_O_4_)_3_]^3−^ in CoFe LDHs. Based on the above results, Figure S2 (Supporting Information) presents two distinct, well‐defined OER model electrocatalysts: brucite‐like layer CoFe‐(CO_3_^2−^)‐LDHs and CoFe‐[Cr(C_2_O_4_)^3^]^3−^‐LDHs.

### 2.2 Electrochemical Properties Toward OER Activity

The OER activity of all samples was assessed using linear sweep voltammograms (LSV) that were acquired in O_2_‐saturated 1.0 m KOH at 1600 rpm using a rotating disk electrode (RDE) (Figure S3A, Supporting Information). The potential is referenced to the RHE and iR corrected for all potential values during each LSV to compensate for the resistance of the solution. The LSV curve for CoFe‐(CO_3_^2−^)‐LDHs exhibited an overpotential of ≈300 mV at 10 mA cm^−2^ (vs RHE) for the OER. The CoFe‐[Cr(C_2_O_4_)_3_]^3−^‐LDHs exhibit a smaller overpotential of 248 mV (vs RHE), which is not only lower than that of CoFe‐(CO_3_^2−^)‐LDHs but also surpasses the performance of the benchmark RuO_2_ (η = 310 mV) and IrO_2_ (η = 402 mV), revealing the effectiveness of [Cr(C_2_O_4_)_3_]^3−^‐ intercalation in boosting OER activity. The CoFe‐[Cr(C_2_O_4_)_3_]^3−^‐LDHs electrocatalyst had superior activity when compared to certain high‐performance CoFe‐based OER catalysts from recent reports, as outlined in Table S1 (Supporting Information). The Figure S3B (Supporting Information) reveals that the Tafel slope of CoFe‐[Cr(C_2_O_4_)_3_]^3−^‐LDHs is 47.5 mV per decade, which is slightly lower than that of CoFe‐(CO_3_^2−^)‐LDHs, which is 81.6 mV per decade, so the former has better kinetics for the OER.

The long‐term electrochemical stability of CoFe‐[Cr(C_2_O_4_)_3_]^3−^‐LDHs reveals high durability over 42 h at an over‐potential of 1.5 V versus RHE for the OER in alkaline media (Figure S3C, Supporting Information). The electrocatalytic property of CoFe‐[Cr(C_2_O_4_)_3_]^3−^‐LDHs degrades negligibly over 3000 CV cycles and electrolysis at 1.5 V, indicating its outstanding long‐term durability. To investigate further the redox peaks of CoFe LDHs before and after the intercalation of [Cr(C_2_O_4_)_3_]^3−^‐, cyclic voltammetry (CV) curves were recorded and plotted in Figure S3D (Supporting Information). The peak at ≈1.17 V is attributed to the oxidation of Co^2+^ sites. In CoFe‐[Cr(C_2_O_4_)_3_]^3−^‐LDHs, the intercalation of [Cr(C_2_O_4_)_3_]^3−^‐ enhanced the energy of formation of Co3+ from Co^2+^, thereby increasing the overall area under the CV curve. Furthermore, the remarkably pronounced Co^2+^ oxidation peak of CoFe‐[Cr(C_2_O_4_)_3_]^3−^‐LDHs indicates that more Co^2+^ sites were oxidized to Co^3+^ and the formation of more CoOOH species, resulting from the intercalation of [Cr(C_2_O_4_)_3_]^3−^‐, which contributes to improved OER performance. The electrochemical double layer capacitance (Cdl), reflecting the electrochemical surface area (ECSA), was estimated from CV curves. As shown in Figure S3E (Supporting Information), the Cdl of CoFe‐[Cr(C_2_O_4_)_3_]^3−^‐LDHs is ≈2.37 times that of CoFe‐(CO_3_^2−^)‐LDHs. The increased ECSA is ascribed to the larger interlayer spacing that is caused by [Cr(C_2_O_4_)_3_]^3−^‐ intercalation and indicates increased exposure to active sites and faster ion exchange (Figure S4A,B, Supporting Information). The stability of OER catalysts holds significant importance for their viable large‐scale practical applications.

In situ/operando electrochemical impedance spectroscopy was performed at different potentials to investigate the electrocatalytic kinetics of the OER and changes in the interphase properties. For CoFe‐(CO_3_^2−^)‐LDHs, the Nyquist plot exhibits an almost steep straight line in the voltage range of 1.05–1.45 V, indicating infinite charge transfer resistance and no occurrence of the OER (Figure S5B, Supporting Information). For CoFe‐[Cr(C_2_O_4_)_3_]^3−^–LDHs, the Nyquist plot shows steep lines in the smaller potential range of 1.05–1.30 V, compared with CoFe‐(CO_3_^2−^)‐LDHs (Figure S5A, Supporting Information). These results demonstrate that the intercalation of [Cr(C_2_O_4_)_3_]^3−^‐ promoted the occurrence of the OER at lower potentials, consistent with the CV curve. Evidently, in the OER, the charge transfer resistance of CoFe‐[Cr(C_2_O_4_)_3_]^3−^‐LDHs is also lower than that of CoFe‐(CO_3_^2−^)‐LDHs when assessed at an applied potential of 1.50–1.55 V on the second semicircle of smaller radius. Figures 3F,S6 (Supporting Information) present Bode phase diagrams that correspond to CoFe‐[Cr(C_2_O_4_)_3_]^3−^‐LDHs and CoFe‐(CO_3_^2−^)‐LDHs, respectively. Two‐phase peaks are observed, with the high‐frequency region originating from the surface double layer capacitance and the low‐frequency region being associated with the non‐uniform charge distribution resulting from surface oxidized species.

Similarly, the oxidation of Co^2+^ to Co^3+^ species for CoFe‐[Cr(C_2_O_4_)_3_]^3−^‐LDHs and CoFe‐(CO_3_^2−^)‐LDHs appear at 1.35 V and 1.45 V, respectively. The intercalation of [Cr(C_2_O_4_)_3_]^3−^‐ changed the electronic structure of CoFe LDHs, reducing the formation energy of Co^3+^(Fe)OOH and the onset potential of the OER from 1.45 to 1.35 V, accompanied by phase transition and high‐frequency oxidation of intrinsic material in the region and the angle associated with the OER interface in the low‐frequency region.

### 2.3 Electronic Structure of As‐Prepared Materials

**Figure 2:**
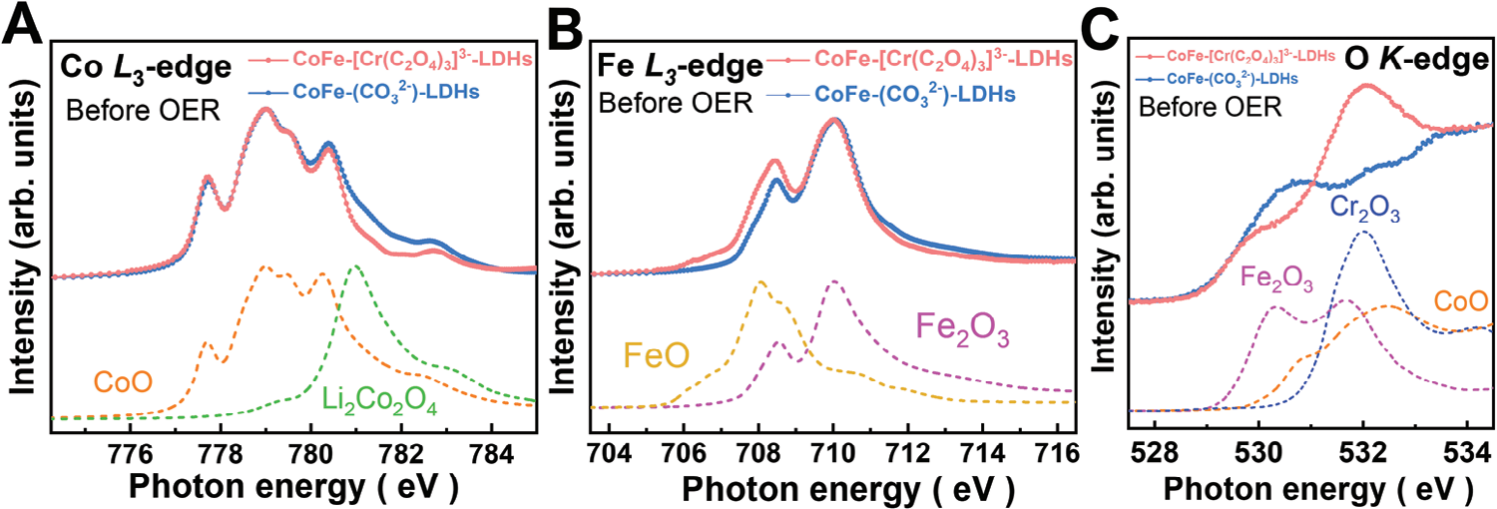
sXAS spectra of CoFe‐(CO_3_^2−^)‐LDHs and CoFe‐[Cr(C_2_O_4_)_3_]^3−^‐LDHs: A) Co L3‐edge, B) Fe L3‐edge and C) O K‐edge. Spectra of Cr_2_O_3_, Fe_2_O_3_, CoO and Li_2_Co_2_O_4_ are included in (A) and (C) as references for high‐spin Co^2+^ and low‐spin Co^3+^, respectively. Spectra of FeO and Fe_2_O_3_ are included in (B) and (C) as references for high‐spin Fe^2+^ and high‐spin Fe^3+^, respectively.^[^ ^54^ ^]^

To understand the origin of the improvement in OER performance from CoFe‐(CO_3_^2−^)‐LDHs to CoFe‐[Cr(C_2_O_4_)_3_]^3−^‐LDHs, the surface electronic structure of the as‐prepared materials is first examined using sXAS at the Co L2,3‐edge and Fe L2,3‐edge. The energy position and multiple spectral features of the Co L2,3‐edge are highly sensitive to the valence state,^[^ ^21^, ^31^ ^]^ spin state^[^ ^47^, ^48^ ^]^ and local environment.^[^ ^49^, ^50^ ^]^ **Figure** **2**A shows the Co L3‐edge sXAS of CoFe‐(CO_3_^2−^)‐LDHs, CoFe‐[Cr(C_2_O_4_)_3_]^3−^‐LDHs, and CoO and Li_2_Co_2_O_4_ as high‐spin Co^2+^ and low‐spin Co^3+^ references, respectively. Both the multiplet spectral features and energy positions of the Co L3‐edge are very similar to those of CoO, indicating that the Co ions of CoFe‐(CO_3_^2−^)‐LDHs and CoFe‐[Cr(C_2_O_4_)_3_]^3−^‐LDHs have a high‐spin Co^2+^ state with octahedral local coordination. The Fe L3‐edge XAS spectra of CoFe‐(CO_3_^2−^)‐LDHs and CoFe‐[Cr(C_2_O_4_)_3_]^3−^‐LDHs (Figure 2B) include two strong peaks at 708.5 and 710.1 eV, resembling those of Fe_2_O_3_, suggesting that the Fe atoms are in the high‐spin Fe^3+^ state. Figure S7 (Supporting Information) displays the Cr L3‐edge XAS of CoFe‐[Cr(C_2_O_4_)_3_]^3−^‐LDHs along with that of Cr_2_O_3_, showing similar spectral features in both materials, revealing the presence of the same Cr^3+^ state. The O K‐edge XAS spectra of CoFe‐(CO_3_^2−^)‐LDHs and CoFe‐[Cr(C_2_O_4_)_3_]^3−^‐LDHs catalysts are investigated and compared with relevant oxide standards, including Fe_2_O_3_, Cr_2_O_3_, and CoO, as shown in Figure 2C. The pre‐edge peaks below 534 eV originate from Co(Fe) 3d and O2p covalent mixing. The energy position of pre‐edge peak shifts to the lower energy with an increase in valence state and atomic number, while intensity increases with increases in valence state and atomic number.^[^ ^35^ ^]^ Based on the L3,2‐edge XAS spectra, the lowest pre‐edge peaks ≈530 eV are attributed to O2p mixing with Fe^3+^ 3d(t2g), while the feature ≈532 eV results from mixing between O2p and Fe3+ 3d (eg), Cr^3+^ and Co^2+^. The electronic structure and even crystal structure of electrocatalysts are known to change under OER conditions.^[^ ^51^, ^52^, ^53^ ^]^ making operando experimental studies crucial for elucidating the mechanism of, and actual active sites in, the OER.

### 2.4 Operando X‐ray Spectroscopic Studies of Electronic and Crystal Structures

**Figure 3:**
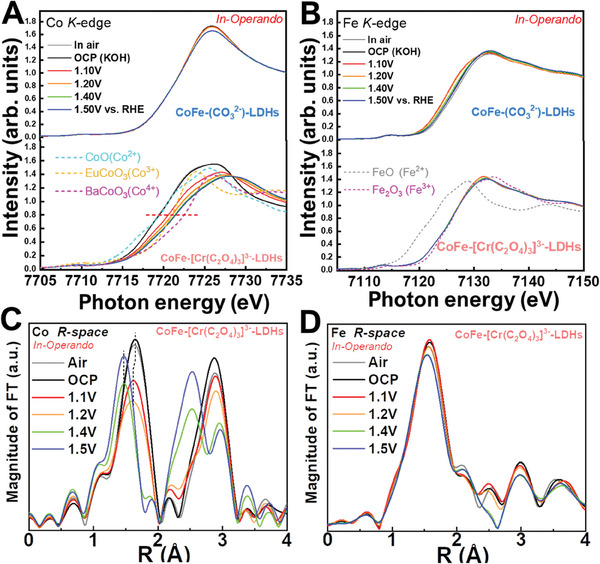
Operando quick‐XAS spectroscopic characterizations. A) Co K‐edge and B) Fe K‐edge XANES spectra of CoFe‐[Cr(C_2_O_4_)_3_]^3−^‐LDHs and CoFe‐(CO_3_^2−^)‐LDHs electrocatalysts at different applied voltages. C) Co K‐edge and D) Fe K‐edge EXAFS spectra of CoFe‐[Cr(C_2_O_4_)_3_]^3−^‐LDHs at various applied potentials.

To identify the catalytic active species in CoFe‐[Cr(C_2_O_4_)_3_]^3−^‐LDHs and CoFe‐(CO_3_^2−^)‐LDHs, operando quick‐XAS studies were conducted to monitor the behavior of the electrocatalyst under electrochemical conditions. The XANES at the 3d transition metal K‐edge is sensitive to the electronic structure,^[^ ^21^, ^25^ ^]^ whereas the EXAFS and XRD spectra offer insights into the crystal structure.^[^ ^54^, ^55^ ^]^ These experimental tools have commonly been used under the operando condition in the past decade.^[^ ^36^, ^56^, ^57^ ^]^ Herein, the charge states of Co and Fe ions in CoFe‐[Cr(C_2_O_4_)_3_]^3−^‐LDHs and CoFe‐(CO_3_^2−^)‐LDHs are first examined under different operando conditions. Figure 3A shows the Co K‐edge XANES spectra of CoFe‐[Cr(C_2_O_4_)_3_]^3−^‐LDHs and CoFe‐(CO_3_^2−^)‐LDHs under various potentials. For comparison, CoO, EuCoO_3_, and BaCoO_3_ are included as references for Co^2+^, Co^3+^ and Co^4+^ states, respectively. Notably, in contrast to CoFe‐(CO_3_^2−^)‐LDHs, which exhibit negligible changes with increasing potentials, a noteworthy observation is that as the applied potential rises, the absorption edge in the spectrum of CoFe‐[Cr(C_2_O_4_)_3_]^3−^‐LDHs shifts to a higher energy, demonstrating an increase in the Co valence state. The energy position, with a normalized intensity of 0.7, of the absorption edge in the sample when exposed to air and under open circuit potential (OCP) conditions is nearly identical and closely resembles that of Co^2+^ reference CoO, suggesting that the as‐prepared material predominantly exists in the Co^2+^ valence state. The Fe K‐edge XANES spectra of CoFe‐[Cr(C_2_O_4_)_3_]^3−^‐LDHs in **Figure** **3**B show almost no shift and are in the same energy position as that of the Fe^3+^ reference Fe_2_O_3_, suggesting that the Co ions are the active sites in CoFe‐[Cr(C_2_O_4_)_3_]^3−^‐LDHs. On the other hand, CoFe‐(CO_3_^2−^)‐LDHs exhibit a different trend under operando conditions. The Fe K‐edge XANES spectra clearly shift to lower energy from Fe^3+^, while the Co K‐edge XANES spectra exhibit nearly no shift (Figure 3A) and closely align with the energy of Co^2+^ reference, strongly indicating that the Fe ions serve as the active sites in CoFe‐(CO_3_^2−^)‐LDHs. Above 1.4 V, when O2 becomes clearly visible in the liquid cell, the energy position moves to higher than that of the Co^3+^ reference EuCoO_3_, suggesting that Co^4+^ may be the active species in CoFe‐[Cr(C_2_O_4_)_3_]^3−^‐LDHs.

**Figure 4:**
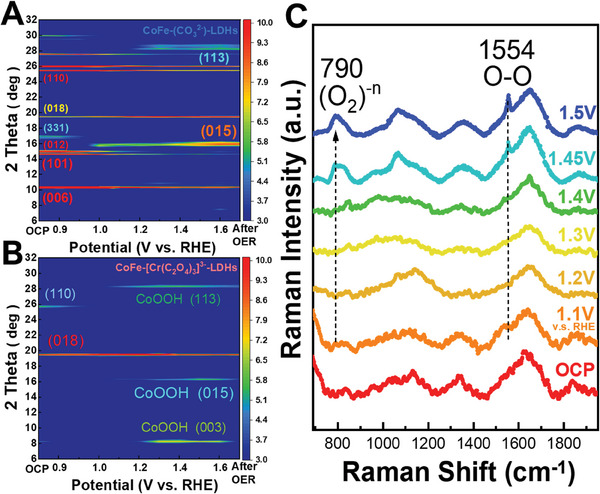
Contour plots of operando X‐ray diffraction signals of A) CoFe‐(CO_3_^2−^)‐LDHs and B) CoFe‐[Cr(C_2_O_4_)_3_]^3−^‐LDHs in aqueous solution of 1 M KOH (pH = 14). Images show diffraction intensity (color‐coded) as a function of voltage, and data were collected at 12 keV. C) In situ/operando Raman characterization of high‐valence Co site during OER.

Subsequently, the atomic structural studies under operando conditions were conducted. The R‐space EXAFS spectra for the Co K‐edge and the Fe K‐edge are presented in Figure 4C,D, respectively, and corresponding cobalt and iron K‐edge k3‐weighted EXAFS spectra of CoFe‐[Cr(C_2_O_4_)_3_]^3−^‐LDHs can be found in Figure S8 (Supporting Information). The first peak at 1.45 Å in Figure 3C is ascribed to Co─O bonds that involve the octahedral Co^2+^‐O shell in air and the OCP, while the peak at 2.8 Å corresponds to the Co─Co(Fe) bond. Two distinct shifts of the Co─O bond at the Co‐K edge at 1.1 and 1.4 V are attributed to the Co^2+^‐to‐Co^3+^ transition and further to the Co^3+^‐to‐Co^4+^ transition, consistent with the XANES spectra discussed earlier. In contrast, the Fe─O bond exhibits only minimal shifts in Figure 3D for CoFe‐[Cr(C_2_O_4_)_3_]^3−^‐LDHs, consistent with the minimal spectral changes observed in the corresponding XANES results. Notably, the decrease in the Co─O bond length from 1.2 to 1.4 V is much larger than that from the OCP to 1.1 V. Given that only a fraction of the Co^3+^ is transformed to Co^4+^, the significant reductions in bond length and splitting of the Co─Co(Fe) bond in Figure 3C suggest a structural transition at 1.4 V. EXAFS results indicate that the intercalation of [Cr(C_2_O_4_)_3_]^3−^‐ effectively accelerates Co pre‐oxidation and self‐reconstruction during the OER.

To further confirm the structural change under OER condition, **Figure** **4**A presents operando XRD patterns of CoFe‐(CO_3_^2−^)‐LDHs at various applied voltages, color‐coded according to diffraction intensity, recorded using 12 keV synchrotron light. At low voltages (below 1.3 V), the main reflections correspond to the brucite‐like layer phase.^[^ ^58^ ^]^ Beyond 1.3 V, the image changes, revealing that the CoO layer transforms into a more active phase with increasing applied potential. The initial state of CoFe‐[Cr(C_2_O_4_)_3_]^3−^‐LDHs in Figure 4B exhibits weak crystallinity due to a large interlayer distance. However, at higher voltage (≈1.4 V), new diffraction lines (003), (015), and (113) emerge, closely resembling those of the β‐CoOOH phase at the electrode surface (Figure S9 (Supporting Information), JCPDS File No. 14–0673).^[^ ^59^ ^]^ This result indicates that the CoO layer transforms into a more active oxyhydroxides phase as the applied potential increases.

**Figure 5:**
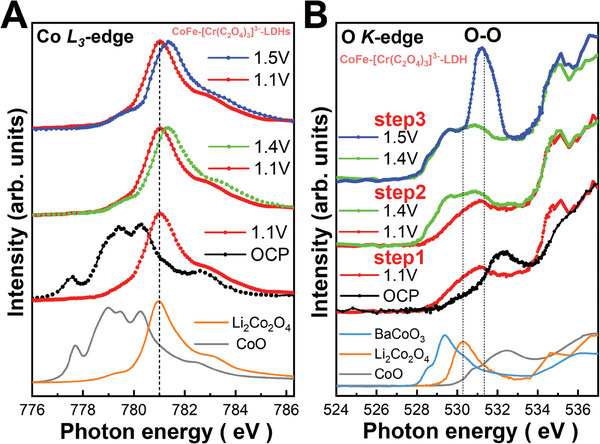
Operando soft‐XAS characterization of high‐valence Co as active site during OER. A) Co L3‐edge and B) O K‐edge of CoFe‐[Cr(C_2_O_4_)_3_]^3−^‐LDHs with references CoO, Li_2_Co_2_O_4_ (low‐spin Co_3+_) and CoOOH.

The CoFe‐[Cr(C_2_O_4_)_3_]^3−^‐LDHs catalyst was further investigated using sXAS at the Co L3‐edges under the operando condition. The illustration of the electrochemical cell is presented in Figure S10 (Supporting Information) in support of operando sXAS at the synchrotron facility during the oxygen evolution reaction. **Figure** **5**A shows the Co L3‐edge XAS of CoFe‐[Cr(C_2_O_4_)_3_]^3−^‐LDHs, along with CoO as a Co^2+^ reference, and LiCoO_3_ as a low‐spin Co^3+^ reference. The energy position and multiplet spectral features at the Co L3‐edge under the OCP (black) are very similar to those of CoO, indicating high‐spin Co^2+^ with CoO6 coordination. From the OCP to 1.1 V (red), the spectrum undergoes a shift to higher energy of more than one eV, exhibiting the line shape and energy position highly resembling that of LiCoO_2_, suggesting the formation of low‐spin Co^3+^. An additional energy shift to 0.25 eV above the main peak of Li_2_Co_2_O_4_ was observed beyond 1.4 V, without alteration in line shape. At 1.5 V, O_2_ gas became clearly visible in the liquid. This phenomenon is highly reminiscent of the transition from low‐spin Co_3+_ to low‐spin Co_4+_ in Li_2_Co_2_O_4_ under the operando condition.^[^ ^35^ ^]^

## 3 Discussion

Until now, above XAS spectroscopic studies on CoFe‐[Cr(C_2_O_4_)_3_]^3−^‐LDHs have been confined to the absorption edge of the metal ion and suggest two‐step transitions, starting with the conversion of Co^2+^ to Co^3+^ and subsequently to Co^4+^, where Co^4+^ is identified as the catalytically active species for the OER akin to Li_2_CoO_4_.^[^ ^35^ ^]^ However, the anionic redox and the formation of O─O bond in conjunction to the 3d6L states occur under the OER condition remains unanswered. Hence, sXAS spectra at the O K‐edge were obtained to yield insights into the transition from Co^2+^ to Co^3+^ and further to Co^4+^. Figure 5B; Figure S11 (Supporting Information) display the O K‐edge of CoFe‐[Cr(C_2_O_4_)_3_]^3−^‐LDHs at various applied voltages, alongside reference materials including CoO, Li_2_Co_2_O_4_, CoOOH and BaCoO_3_, representing Co^2+^, Co^3+^, and Co^4+^ states. The first valence state transition from the OCP to 1.1 V is evident in Figure 5B. A new lower‐energy pre‐edge peak emerges, which can be attributed to the Co^3+^ 3d‐O2p state, as elucidated in the previous analysis of the Co L L3‐edge. However, the line shape of CoFe‐[Cr(C_2_O_4_)_3_]^3−^‐LDHs appears significantly broader in comparison to LiCoO_2_, primarily due to the presence of oxyhydroxides phase similar to CoOOH.^[^ ^4^ ^]^

At the voltage above 1.1 V, two new pre‐edge peaks appear at 528.5 and 529.5 eV, as depicted in Figure S12 (Supporting Information), and their spectral weights rise up to 1.4 V. The peaks at the lower and higher energies are assigned to t2g and eg when compared to Li0.66CoO_2_, and the overall spectral feature is very similar to that of Li_2_Co_2_O_4_ under the OER condition,^[^ ^35^ ^]^ which is consistent with the findings from the Co K‐edge XANES, Co L3‐edge XAS, and operando Raman spectra as presented in Figure S13 (Supporting Information). Notably, the spectral weight of 3d6L state, represented as the electron hole (h+) on O^2−^,^[^ ^27^ ^]^ is similar to that of Li_2_Co_2_O_4_, as shown in Figure S13 (Supporting Information), prior to the release of O_2_ in the liquid cell. To further support the argument, isotopic study is performed. The in situ Raman spectroscopy in 1 m KO(18)H electrolyte is carried out. As shown in Figure S14 (Supporting Information), the peak at 790 and 1554 cm^−1^ shift to a lower wavenumber of 746 m^−1^ and 1468 cm^−1^ corresponding to (O_2_)‐n and O─O bonds.

Further raising the voltage from 1.4 to 1.5 V not only marginally enhances the spectral weight of the 3d6L state but also results in a substantial peak at 531.5 eV, as shown in Figure 5B. The oxidization of Co and Fe ions can be ruled out as the cause of this substantial peak at 531.5 eV, as such oxidation would lead to an increase in the spectral weight below 530 eV. The possibility of this substantial peak being associated with O_2_ gas can also be excluded, as the pre‐edge peak of O_2_ gas exhibits an intrinsic narrow peak width and would not account for such a large spectral weight owing to the relatively low O_2_ content. Nonetheless, this spectral feature is recognized as being related to the O─O bond in battery materials.^[^ ^27^ ^]^ Bergmann et al. provided detailed calculations,^[^ ^4^ ^]^ but did not clearly observe this spectral feature. Our work provides the first experimental evidence supporting the theoretical prediction. (Figure S15, Supporting Information) This assignment is further supported by the Operando Raman spectra in Figure 4C, which revealed two new Raman bands with increasing the applied voltages. One peak is observed at 1555 cm^−1^, contributed to O2, while another peak appears ≈790 cm^−1^, corresponding to the O─O bond,^[^ ^27^ ^]^ the latter band falls within the range of 743 cm^−1^ for peroxide (O_2_)^2−^ and 1108 cm^−1^ for superoxide (O_2_)^1−^. The Operando O K‐edge XAS and the operando Raman spectra collectively demonstrate the simultaneous formation of the O─O bond and O_2_. The electronic structural response to the applied voltage in CoFe‐[Cr(C_2_O_4_)_3_]^3−^‐LDHs differs significantly from that in Li_2_Co_2_O_4_, where O_2_ is released without the emergence of a peak associated with the O─O bond at 531.5 eV. A two‐step change in the electronic structure has been observed in Cu_2_O^[^ ^32^ ^]^ and UCoO_4_,^[^ ^60^ ^]^ however, no three‐step change has been reported previously.

A test of the electrochemical reversibility of the substantial peak at 531.5 eV was conducted. The voltage was alternated between 1.4 and 1.5 V, resulting in the appearance of the substantial peak at 1.5 V and its disappearance at 1.4 V, as shown in Figure S12 (Supporting Information). Thus, it is concluded that the third‐step transition from 1.4 to 1.5 V is reversible, whereas the first and second‐step transitions are irreversible. The ex situ Co L3‐edge and O K‐edge of CoFe‐[Cr(C_2_O_4_)_3_]^3−^‐LDHs were measured after the OER, as presented in Figure S11 (Supporting Information). Notably, after the ex situ OER, the substantial peak at 531.5 eV is no longer observed. However, the Co^4+^‐related spectral feature persists in the O K‐edge XAS and the Co L3‐edge XAS, similar to those measured at 1.4 V under the operando condition, further supporting the significance and reversibility of the third‐step transition.

## 4 Conclusion

In conclusion, different operando X‐ray spectroscopic techniques were used to observe the formation of O─O by three‐step oxidation of a CoFe‐(CO_3_^2−^)‐LDHs catalyst after the intercalation of [Cr(C_2_O_4_)_3_]^3−^‐ during the oxidation of water. The first two steps involve irreversible oxidation of Co^2+^ to Co^3+^ and further to Co^4+^, accompanied by structural transformation. In contrast, the third step notably discovers for the first time that the formation of the O─O band and O_2_ gas has been identified in the operando O K‐edge XAS and Raman spectra. This process differs significantly from that of LDHs lacking [Cr(C_2_O_4_)_3_]^3−^‐ intercalation, where the oxidation primarily converts Fe^3+^ to Fe^4+^ without inducing structural transformations. Compared to the pure CoFe‐LDHs, which exhibit a 300 mV overpotential at 10 mA cm^−2^ in pure LDHs, the CoFe‐[Cr(C_2_O_4_)_3_]^3−^‐LDHs catalyst demonstrates a significantly lower overpotential of 248 mV at 10 mAcm^−2^, a small Tafel slope of 47.5 mVdec^−1^ and excellent operational stability. Given that the Co4+(oxyhydr)oxide was observed in both CoFe‐[Cr(C_2_O_4_)_3_]^3−^‐LDHs and Co(OH)2,^[^ ^61^ ^]^ the exclusive presence of O─O bonds in the former can be assigned to the pronounced structural shrinkage, which is critical in achieving high OER activity due to the creation of a shorter reaction pathway.^[^ ^62^ ^]^

## 5 Experimental Section

### Sample Preparation

In this experiment, (Co:Fe = 3:1) CoFe‐[Cr(C_2_O_4_)_3_]^3−^‐LDHs materials were prepared by the hydrothermal method. Mixed solution 1 comprised FeCl3·6H2O (0.005 mol) and CoCl2·6H2O (0.015 mol) in 50 mL of purified deionized water. Solution 2 comprised Na_2_CO_3_ (0.036 mol) and K_3_[Cr(C2O4)3]·3H2O (0.004 mol) in 40 mL of deionized water. Solutions 1 and 2 were then vigorously mixed. The homogeneous slurry thus formed was transferred to a stainless‐steel Teflon‐lined autoclave and heated at 100 °C for 20 h. It was then left to cool naturally to room temperature. The obtained precipitate was washed several times using deionized water and finally dried at 60 °C. Except for the addition of solution 2, the rest of the synthetic process was the same as for CoFe‐[Cr(C_2_O_4_)_3_]^3−^‐LDHs above.

### Characterization

The morphologies of the electrocatalysts were characterized by transmission electron microscopy (TEM, Tecnai G2 F20) and scanning electron microscopy (SEM, Hitachi, S‐4800). Inductively coupled plasma emission spectrometer mapping ((ICP, PerkinElmer 8300, PerkinElmer, Inc., USA) was used for elemental analysis. Fourier transform infrared (FTIR) spectroscopy was conducted using a Bruker Tensor II spectrometer (BRUKER Co., Bremen, Germany).

### Electrochemical Measurements

All electrochemical measurements were made at 298 K on a CHI 600E electrochemical workstation using a typical three‐electrode system. A saturated calomel electrode (SCE) was used as the reference electrode; a graphite rod was used as the counter electrode, and the electrocatalyst was dropped on glass with a carbon electrode (5 mm in diameter, 0.196 cm^2^) with a loading of 0.203 mg cm^−2^ to form the working electrode. Prepare the working electrode by dispersing 5 µL of the catalyst ink. The catalyst ink was obtained by dispersing 10 mg of LDHs powder and 10 mg carbon black into 1 ml absolute ethanol and 0.1 ml 5 wt.% Nafion solution. The mixture was then gently sonicated to form the homogeneous catalyst ink. All linear sweep voltammetry (LSV) curves were recorded at a scan rate of 5 mV s^−1^ and corrected by removing iR dips in 1.0 m KOH. All OER potentials were calibrated with reference to the RHE scale (0.9335 V in 0.1 m KOH). In a three‐electrode system, impedance measurements were performed across a frequency range from 1 MHz to 0.1 Hz, using 1.0 m KOH and an Autolab electrochemical workstation (Autolab PGSTAT302N, Metrohm‐Autolab BV, The Netherlands).

### Operando Quick‐XAS Measurements

Operando quick‐XAS XANES and EXAFS measurements were made at the Taiwan Photon Source (TPS), NSRRC. The Quick‐Scanning XAS beamline, BL 44A, was a hard X‐ray bending magnet beamline that covers the energy range 4.5–34 keV; it supports fast scanning for making Operando time‐resolved XAFS measurements.^[^ ^63^ ^]^ A full Quick‐Scanning XAFS spectrum can be obtained in less than 100 ms over an energy range of more than 1000 eV. The XAS measurements at the Fe K‐edge (7112 eV) and Co K‐edge (7709 eV) were made using transmission mode. The regular XAS at Fe and Co K‐edge were also measured and confirmed at TPS 32A. The energy resolution of Fe K‐edge and Co K‐edge XANES was set to ≈0.35 eV. XANES data were treated using standard procedures, including background subtraction and normalization of the edge height. EXAFS uses the k3‐weighted oscillation Fourier transformation to determine the local atomic environments of Fe and Co atoms. In addition, the transmission mode of EXAFS enables easy in situ measurement. The ease of data collection enables Quick‐XAFS to capture the transient states during the OER reaction, with barely any interference from oxygen bubbles released form the catalyst surface.

In this experiment, CoFe‐[Cr(C_2_O_4_)_3_]^3−^‐LDHs and CoFe‐(CO_3_^2−^)‐LDHs powders were added dropwise to 50 µL 5 wt% Nafion solution to form homogeneous suspensions. Ultrasonic dispersion was conducted for 20 min. Data were acquired for 0.5 s per spectrum. To ensure that the working catalysts were in a steady state during in situ characterization, each characterization was followed by five min of electrolysis. Quick‐XAFS scans generate a large volume of data. The data were compiled on‐site using a customized data processing program, allowing the user to export each EXAFS scan as a single file or combine several consecutive scans into one file. In the current analysis, the output data were obtained by combining 240 scans, which covered a time window of 120 s. The data were then processed using ATHENA for energy alignment and normalization.^[^ ^64^ ^]^

### Operando soft‐XAS Measurements

The sXAS experiments at the O K‐edge, Fe L3‐edge and Co L3‐edge were performed at beamline TLS BL‐11A in TFY mode at NSRRC. Single crystals of NiO, Fe_2_O_3_ and CoO were purchased from Matek Material Technologie & Kristalle GmbH. The spectra of these single crystals were concurrently recorded in an independent ultrahigh vacuum chamber in total electron yield (TEY) mode to calibrate the energies for the measurements at the O K‐edge, Fe L3‐edge and Co L3‐edges, respectively. For Operando sXAS, the CoFe‐[Cr(C_2_O_4_)_3_]^3−^‐LDHs catalyst powder was dispersed in ethanol and deionized water and then sonicated for 30 min. The ink was then drop‐cast on carbon paper with a loading mass of 0.3 mg cm^−2^ for use in subsequent ex situ sXAS experiments. For the operando experiments, the ink was dropped on a thin membrane window (100 nm–thick silicon nitride with an area of 1 × 1 mm^2^, coated with 3 nm Ti/10 nm Au from Silson Ltd.) with a loading mass of ≈1 mg cm^−2^. This window was used both as the working electrode and to separate the liquid from the ultrahigh vacuum environment.

The operando sXAS experiments were conducted using an in situ electrochemical liquid cell with three (working, reference, and counter) electrodes that were controlled using a VersaSTAT 4 potentiostat from Princeton Applied Research. Two platinum wires were used as the reference and counter electrodes. Here, a Pt pseudo reference electrode was used because space in the electrochemical cell was limited and the potential was calibrated to RHE following the procedure that was described by Kasem and Jones.^[^ ^65^ ^]^ Freshly prepared O_2_‐saturated 1.0 m KOH was used as the electrolyte, and the electrochemical liquid cell system contained a liquid pump, an inlet, and an outlet tube to allow it to flow. The diagram in Figure S10 (Supporting Information) illustrates the concept of the design for the in situ electrochemical cell. Fluorescence yield (FY) mode was used to collect the absorption signal for the operando measurement.

## Conflict of Interest

The authors declare no conflict of interest.

## Author Contributions

Y.C.H. and Y.W. contributed equally to this work. Y.C.H., Y.W., S.W., W.C.C. and C.L.D. conceived and designed the project. Y.W. and Y.C.H. contributed to the sample preparation and analyzed the overall experimental data under the supervision of S.W., Y W. and C.L.D.; Y.R.L., C.L.C. and J.L.C. supported operando hard XAS experiments. Y.C.H., C.L.D. H.J.L., C.T.C. and Z.H. supported the liquid cell for sXAS experiments and contributed to soft‐XAS experiments and data analyses. C.J., J.Z., and L.Z. supported operando Raman experiments and O18 operando Raman experiments. Y.C.H. and C.L.D. wrote the manuscript. All the other authors discussed the results and assisted during the manuscript preparation. C.L.D. was responsible for the project management.

## Supporting information

Supporting Information

## Data Availability

The data that support the findings of this study are available from the corresponding author upon reasonable request.
