# Supplementary material for: Direct Identification of O─O Bond Formation Through Three‐Step Oxidation During Water Splitting by Operando Soft X‐ray Absorption Spectroscopy
Source: Adv Sci (Weinh). 2024 Aug 1;11(40):2401236. doi: 10.1002/advs.202401236 (PMC11515896; doi:10.1002/advs.202401236)
Supplement: Supplementary file 1 — Supporting Information [file ADVS-11-2401236-s001.docx]

**Supporting Information**

**Direct Identification of O-O Bond Formation Through Three-step Oxidation During Water Splitting by Operando Soft X-ray Absorption Spectroscopy**

Yu-Cheng Huang,^a,d#^ Yujie Wu,^b,#^ Ying-Rui Lu,^a^ Jeng-Lung Chen,^a^ Hong-Ji Lin,^a^ Chien-Te Chen,^a^ Chi-Liang Chen,^a^ Chao Jing,^c^ Jing Zhou,^c^ Linjuan Zhang,^c^ Yanyong Wang,^b^ Wu-Ching Chou,^d,*^ Shuangyin Wang,^b,*^ Zhiwei Hu,^e,*^ and Chung-Li Dong,^g,*^

1. *National Synchrotron Radiation Research Center, Hsinchu 30076, Taiwan*
2. *State Key Laboratory of Chemo/Bio-Sensing and Chemometrics, College of Chemistry and Chemical Engineering, Advanced Catalytic Engineering Research Center of the Ministry of Education, Hunan University, Changsha 410082, China*
3. *Key Laboratory of Interfacial Physics and Technology, Shanghai Institute of Applied Physics, Chinese Academy of Sciences, Shanghai 201800, China*
4. *Department of Electrophysics, National Yang Ming Chiao Tung University, Hsinchu City 300093, Taiwan*
5. *Max-Planck-Institute for Chemical Physics of Solids, Dresden 01187, Germany*
6. *Research Center for X-ray Science & Department of Physics, Tamkang University, New Taipei City 25137, Taiwan*

*[#] These authors contributed equally to this work.*

*E-mail address: shuangyinwang@hnu.edu.cn (Shuangyin Wang), Zhiwei.Hu@cpfs.mpg.de (Zhiwei Hu),* *cldong@mail.tku.edu.tw (Chung-Li Dong),* [wcchou957@nycu.edu.tw](mailto:wcchou957@nycu.edu.tw) (Wu-Ching Chou)


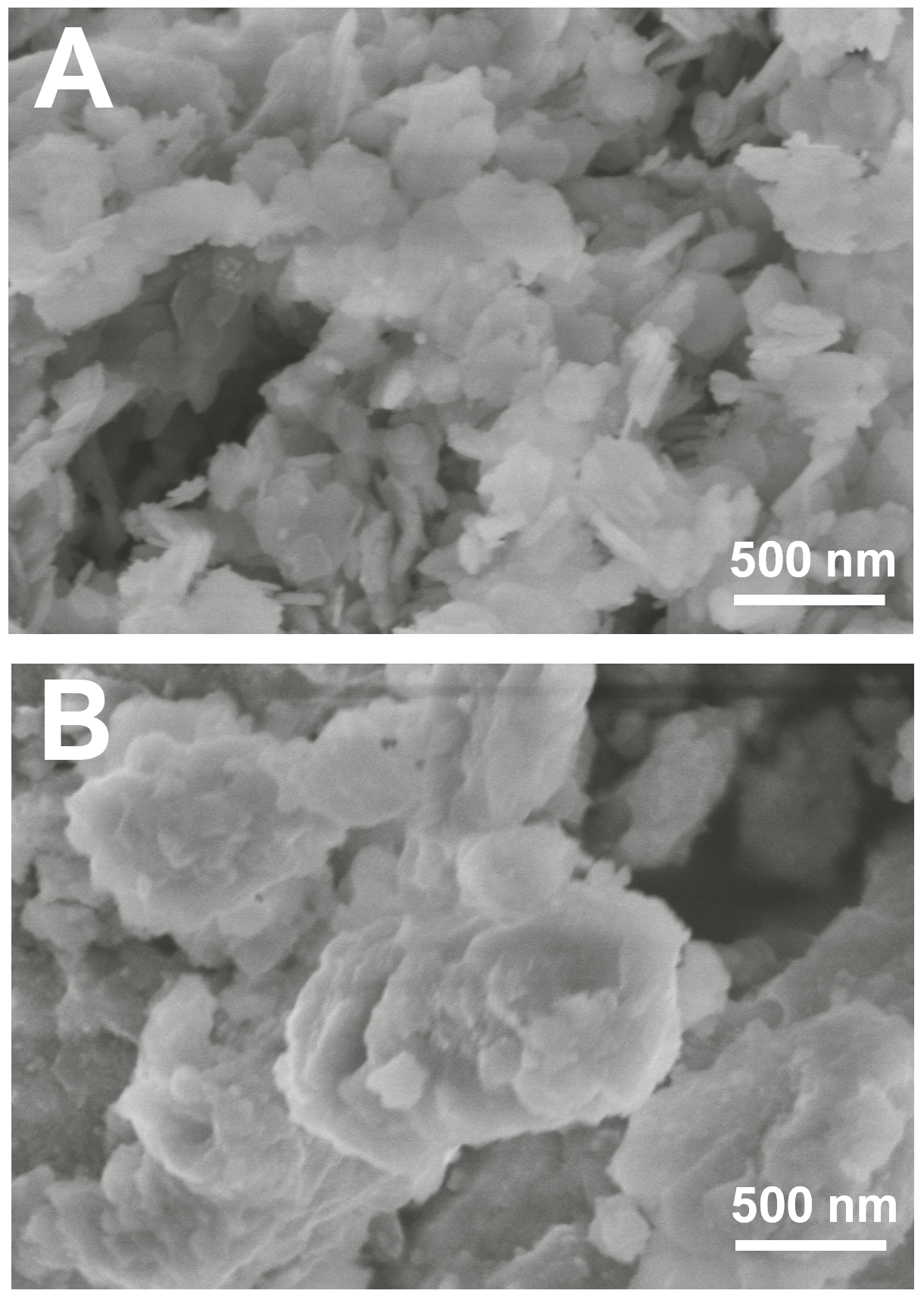


Figure S1. (**A)** SEM image of CoFe-(CO_3_^2-^)-LDHs. (**B)** SEM image of CoFe-[Cr(C_2_O_4_)_3_]^3-^-LDHs.


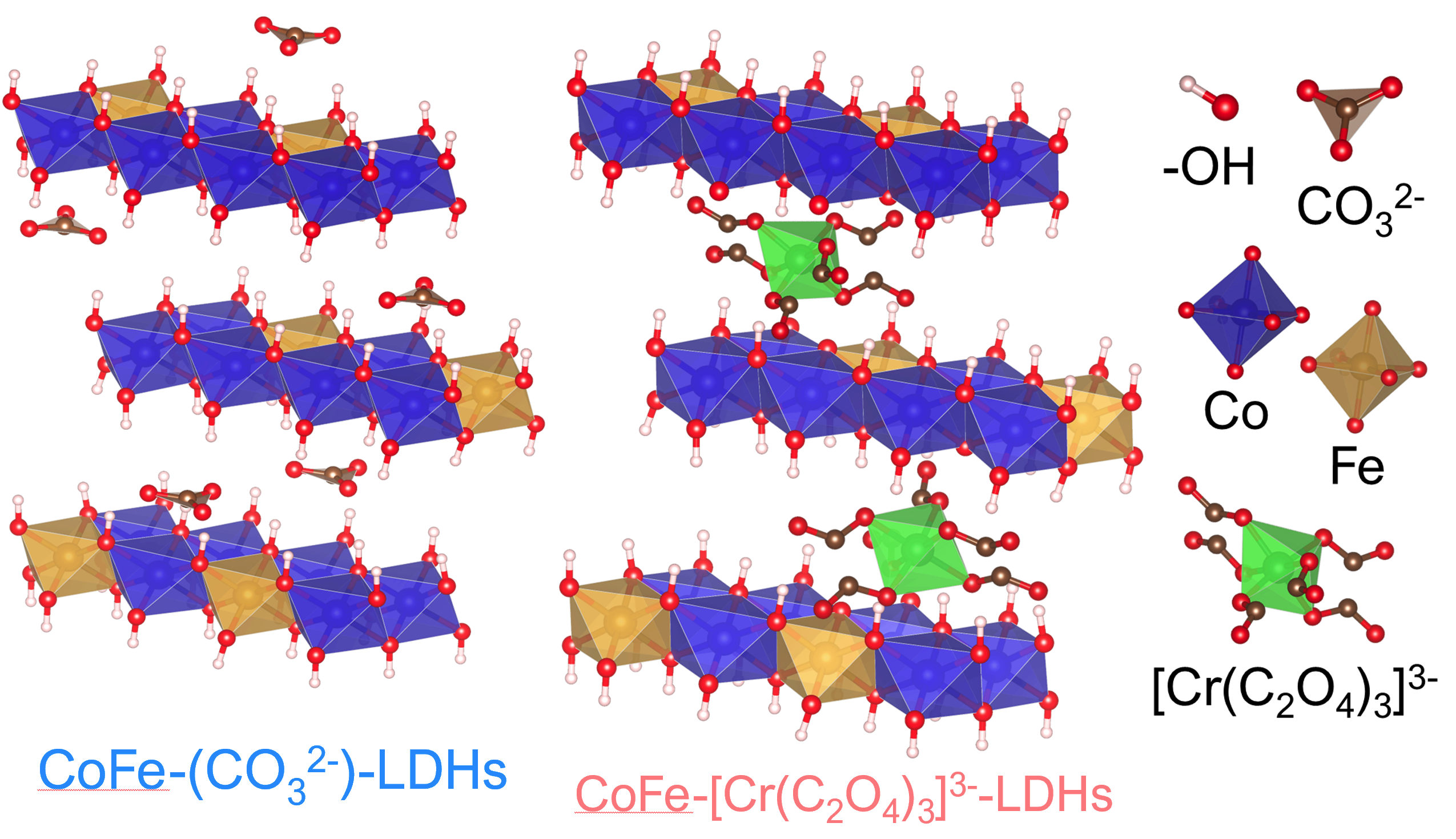


Figure S2. Prepared crystal structure. Layers with four metal atoms each show a fully protonated form of brucite-like CoFe-(CO_3_^2-^)-LDHs with intercalated carbonate anions (randomly distributed), and CoFe-[Cr(C_2_O_4_)_3_]^3-^-LDHs with intercalated chromate anions.


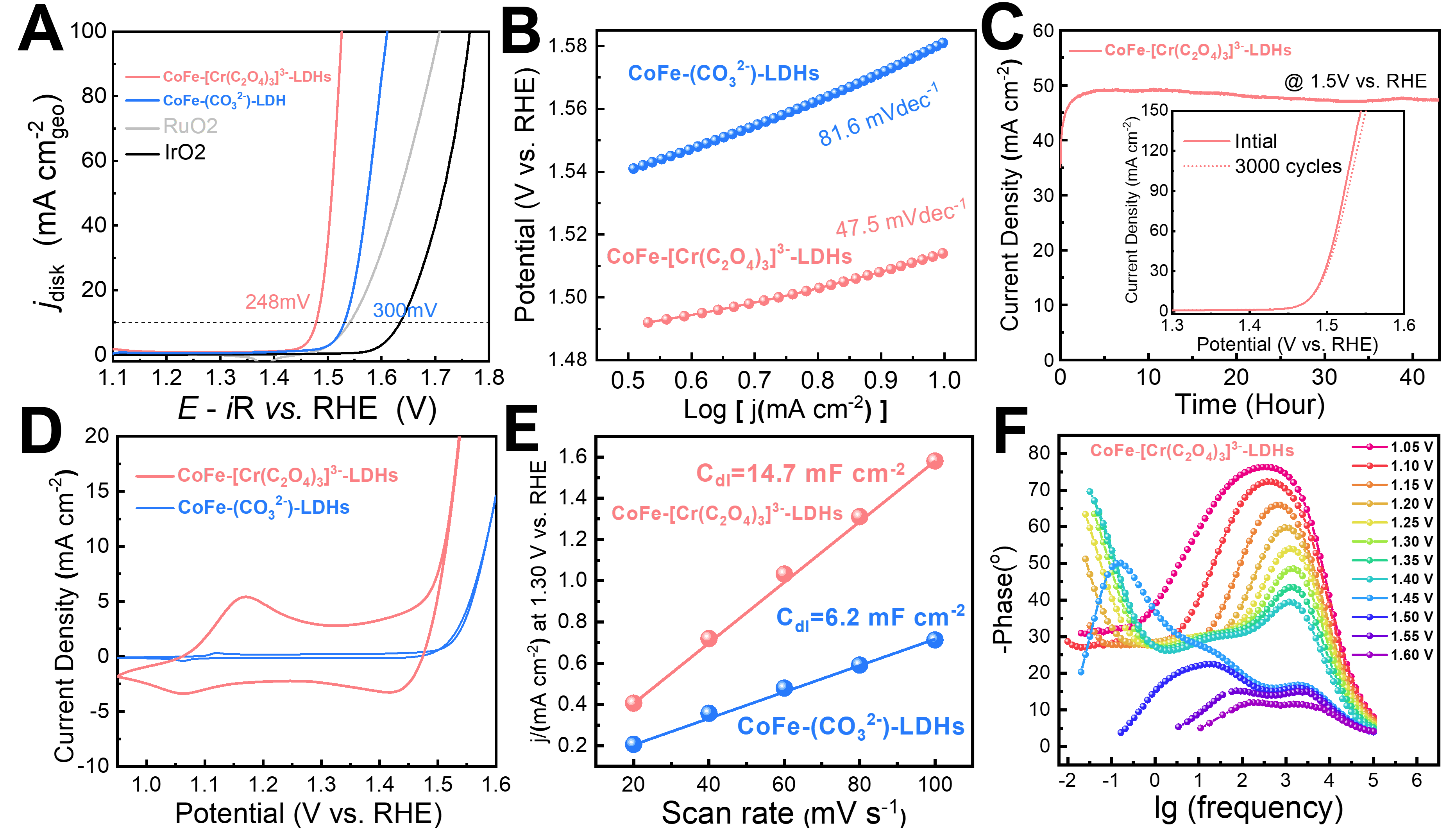


Figure S3. **(A)** OER polarization curves of CoFe-(CO_3_^2-^)-LDHs and CoFe-[Cr(C_2_O_4_)_3_]^3-^-LDHs substrate in KOH solution (1 M). **(B)** Tafel plots of CoFe-(CO_3_^2-^)-LDHs and CoFe-[Cr(C_2_O_4_)_3_]^3-^-LDHs. **(C)** Long-term electrochemical stability of CoFe-[Cr(C_2_O_4_)_3_]^3-^-LDHs was measured at an over-potential of 1.5 V vs. RHE. The polarization curves of CoFe-[Cr(C_2_O_4_)_3_]^3-^-LDHs were obtained before and after 3000 CV cycles. (inset). **(D)** Cycling voltammetry curves of CoFe-(CO_3_^2-^)-LDHs and CoFe-[Cr(C_2_O_4_)_3_]^3-^-LDHs. **(E)** Calculated electrochemical double-layer capacitance at various scan rates for as-prepared catalysts. **(F)** Bode phase plots for CoFe-[Cr(C_2_O_4_)_3_]^3-^-LDHs in 1.0 M KOH at various applied potentials.


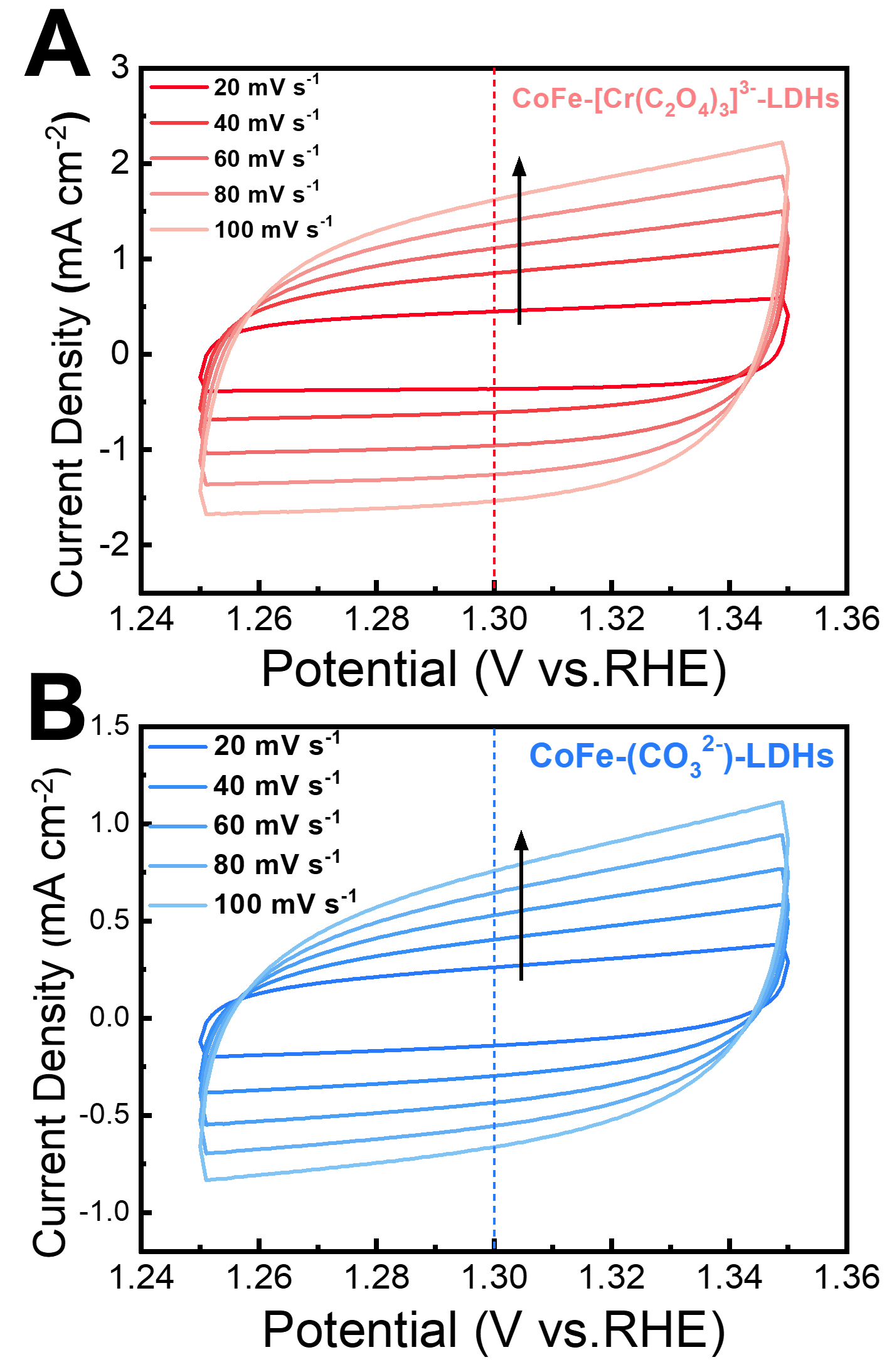


Figure S4. CV curves at varied scan rates for **(A)** CoFe-[Cr(C_2_O_4_)_3_]^3-^-LDHs and **(B)** CoFe-(CO_3_^2-^)-LDHs.


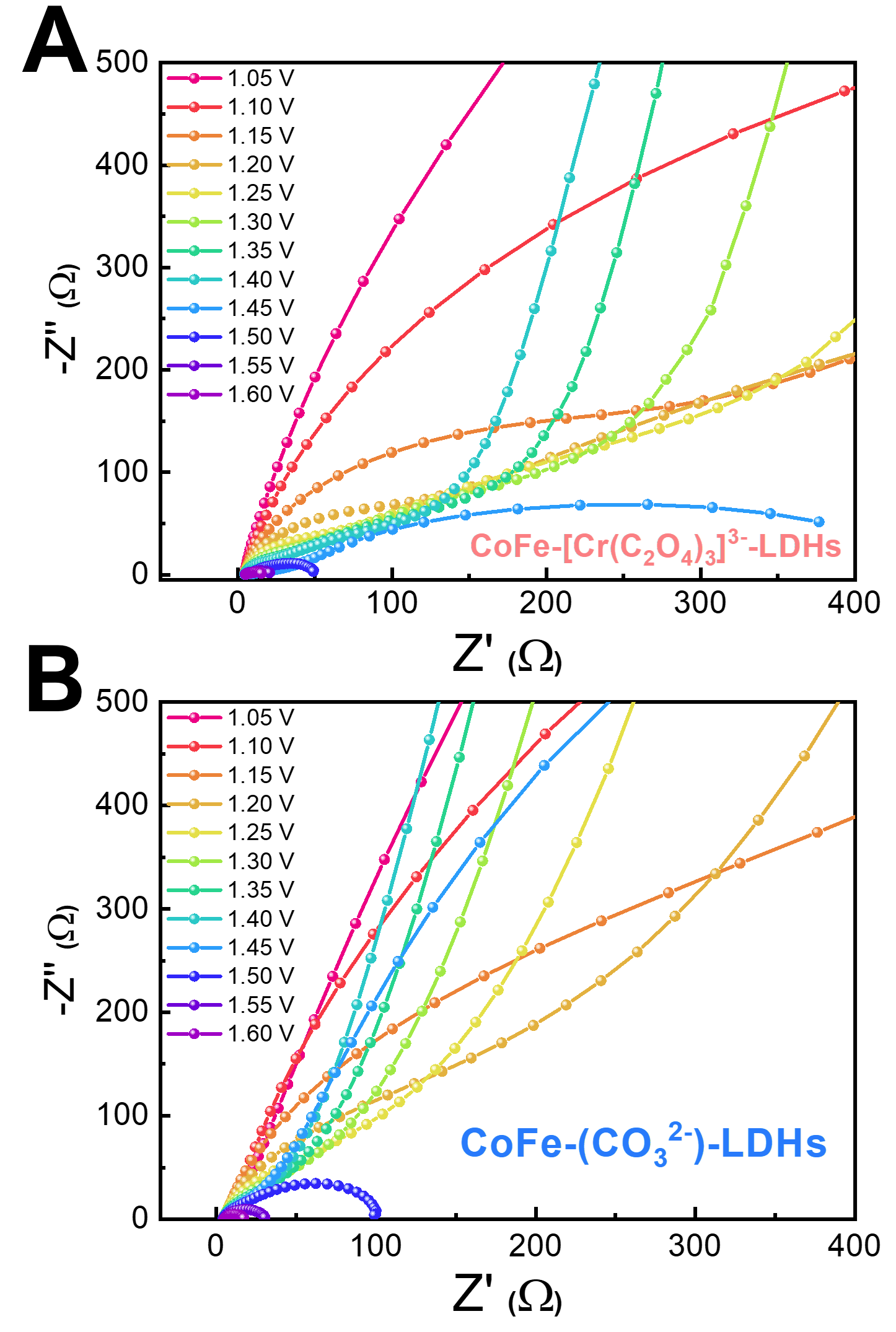


Figure S5. Voltage-independent Corresponding Nyquist plots for **(A)** CoFe-[Cr(C_2_O_4_)_3_]^3-^-LDHs and **(B)** CoFe-(CO_3_^2-^)-LDHs. in 1.0 M KOH.


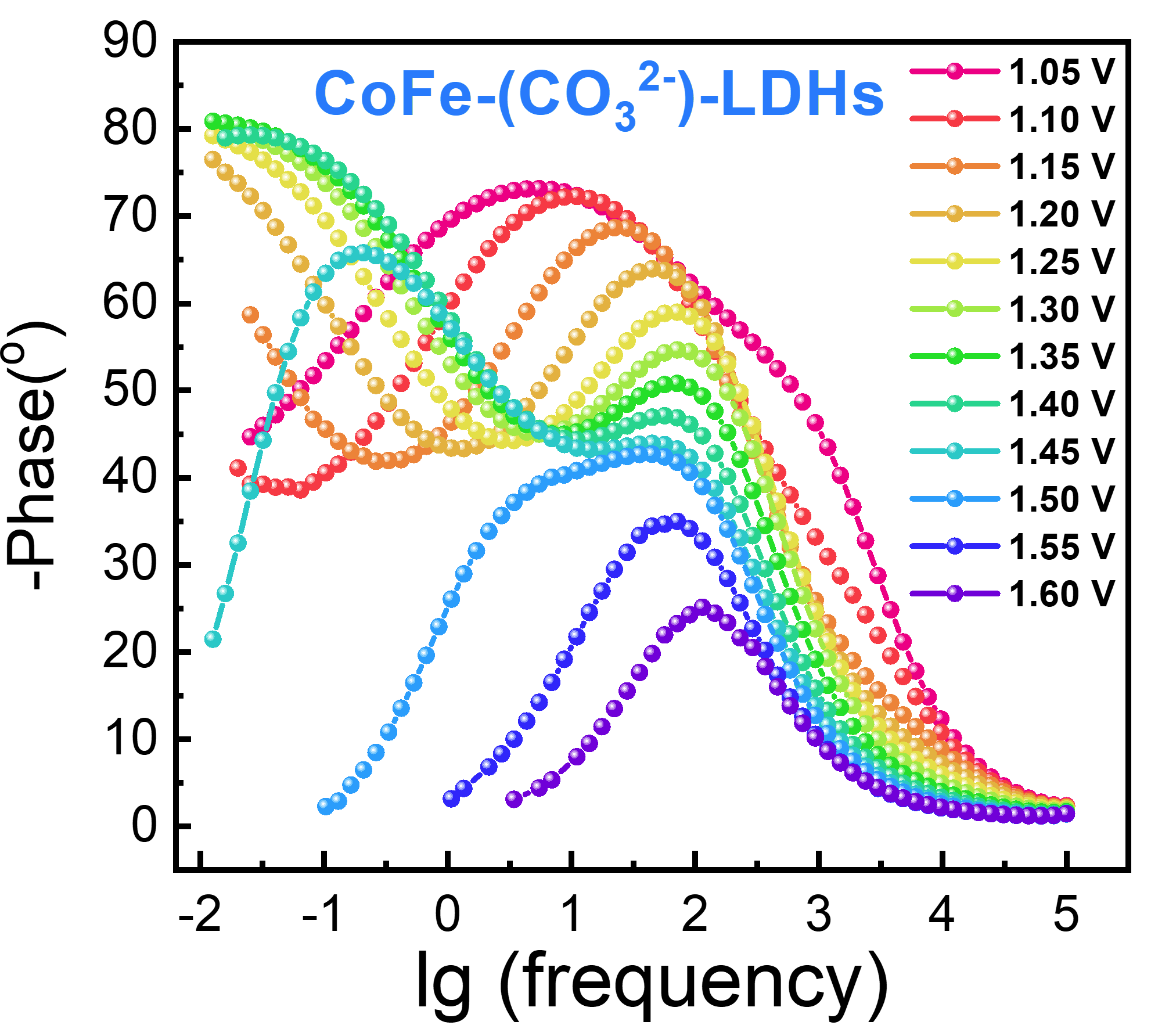


Figure S6. Corresponding Bode phase plots for CoFe-(CO_3_^2-^)-LDHs in 1.0 M KOH at various applied potentials.


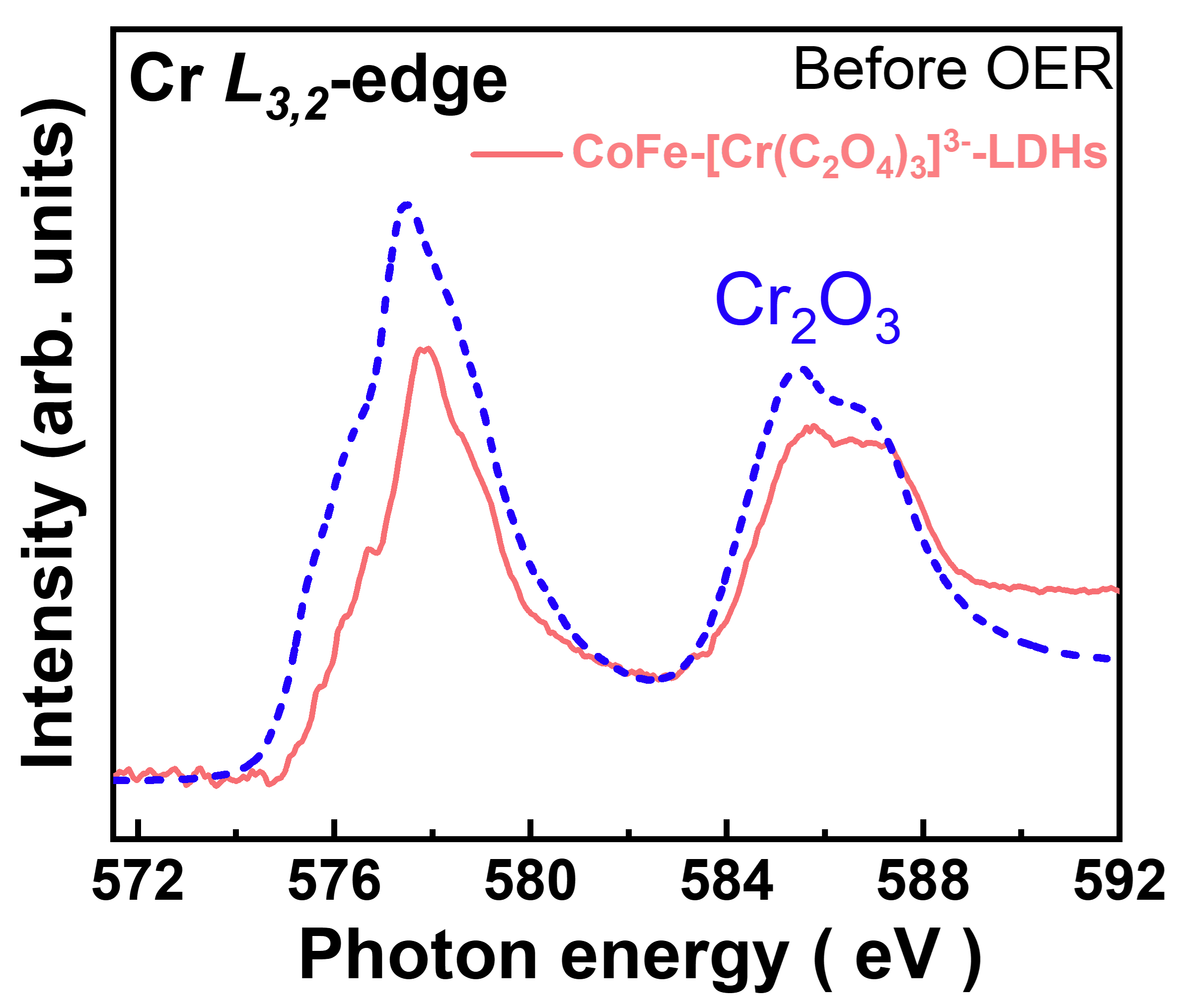


Figure S7. The sXAS measurements on the Cr *L_3,2_*-edge of CoFe-[Cr(C_2_O_4_)_3_]^3-^-LDHs. The Cr *L_3,2_*-edge along with Cr_2_O_3_(Cr^3+^) references.


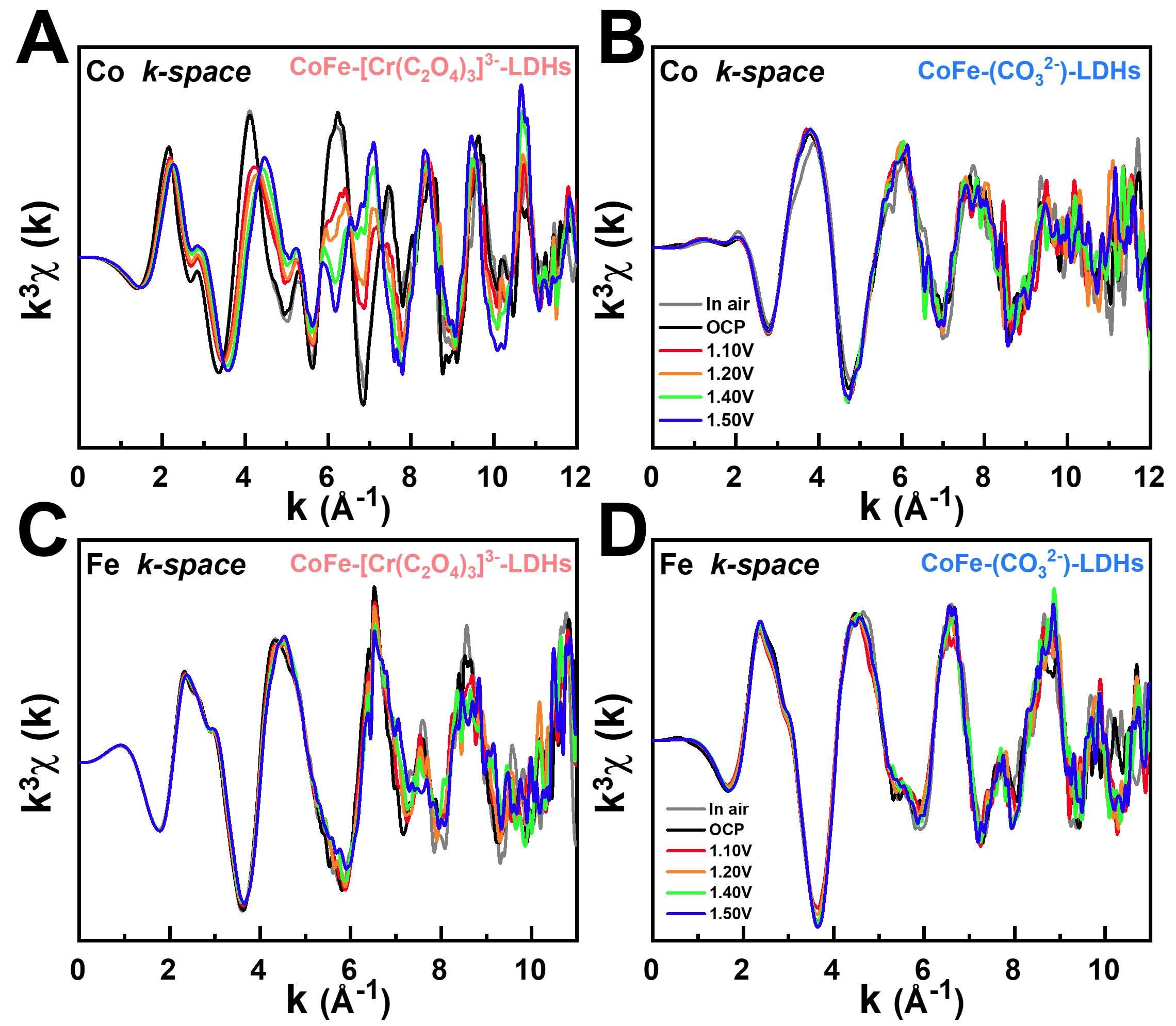


Figure S8. Summary of k^3^-weigted Co *K*-edge EXAFS spectra of **(A)** CoFe-[Cr(C_2_O_4_)_3_]^3-^-LDHs and **(C)** CoFe-(CO_3_^2-^)-LDHs, plotted as the amplitude and the real part. Summary of k^3^-weigted Fe *K*-edge EXAFS spectra of **(B)** CoFe-[Cr(C_2_O_4_)_3_]^3-^-LDHs and **(D)** CoFe-(CO_3_^2-^)-LDHs, plotted as the amplitude and the real part.


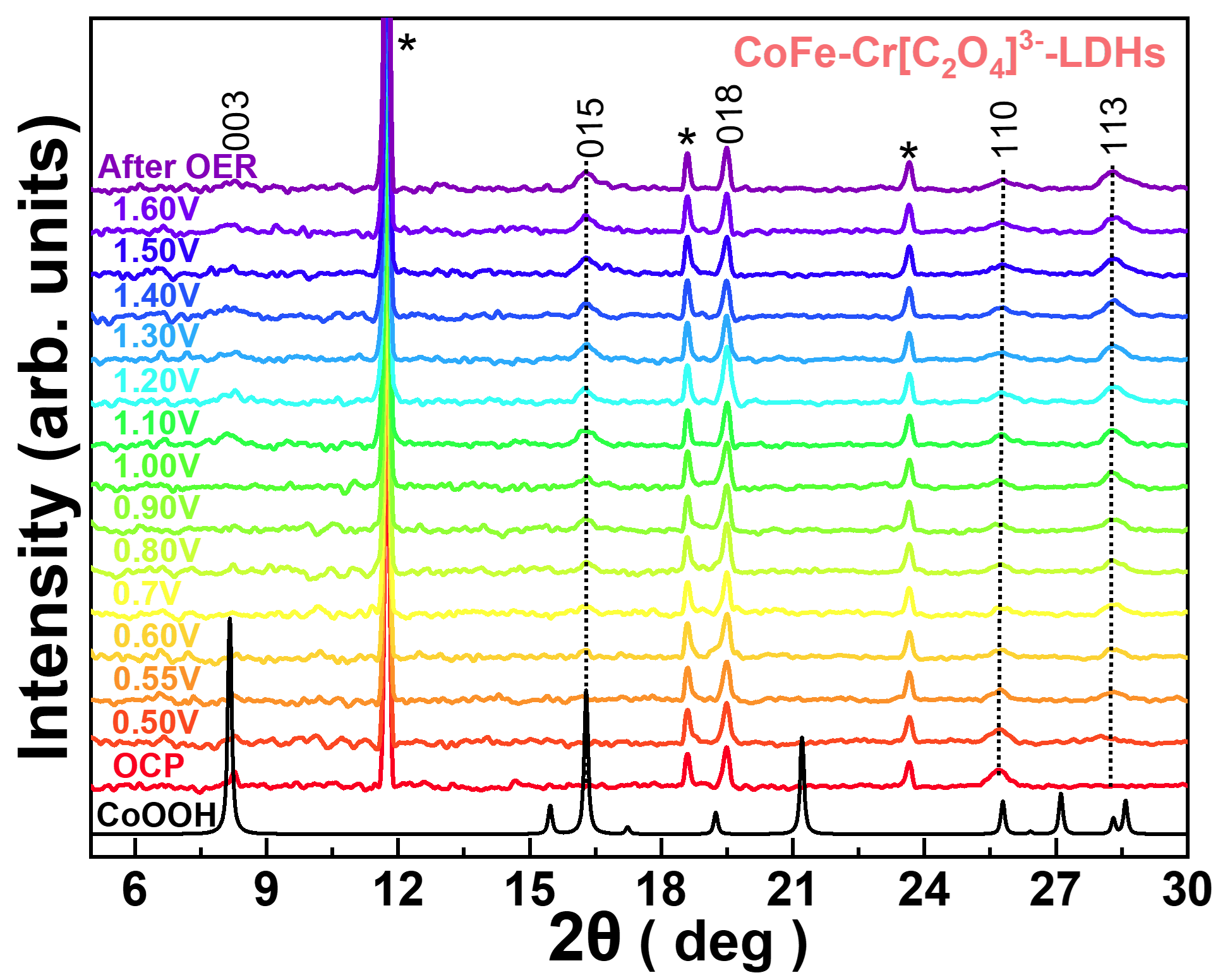


Figure S9. *Operando* XRD signals of CoFe-[Cr(C_2_O_4_)_3_]^3-^-LDHs in an aqueous solution containing 1 M KOH (pH = 14), standard references ($\beta$-CoOOH)^[1]^.


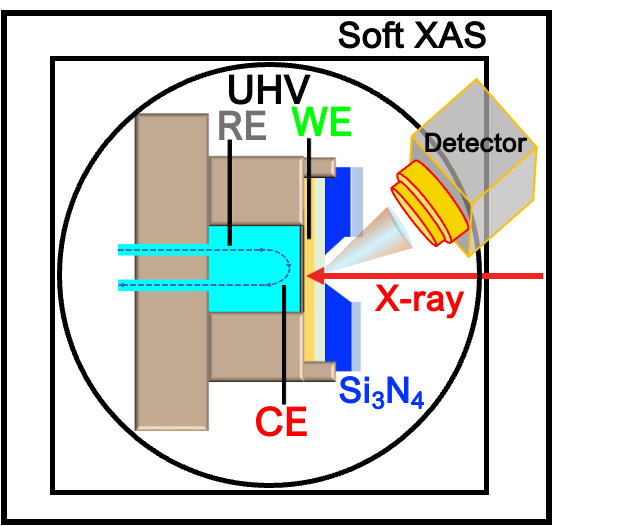


Figure S10. Illustration of the in situ electrochemical cell for the *operando* soft X-ray absorption spectroscopy using synchrotron X-rays during the oxygen evolution reaction.

The design of the electrochemical cell is based on Guo`s three-electrode design at the Advanced Light Source, Lawrence Berkeley National Laboratory,^[2]^ which demonstrated the first *in-situ* electrochemical soft-XAS study to monitor the electrochemical corrosion of copper in NaHCO_3_ solution. Herein, the key concept of the design is illustrated.


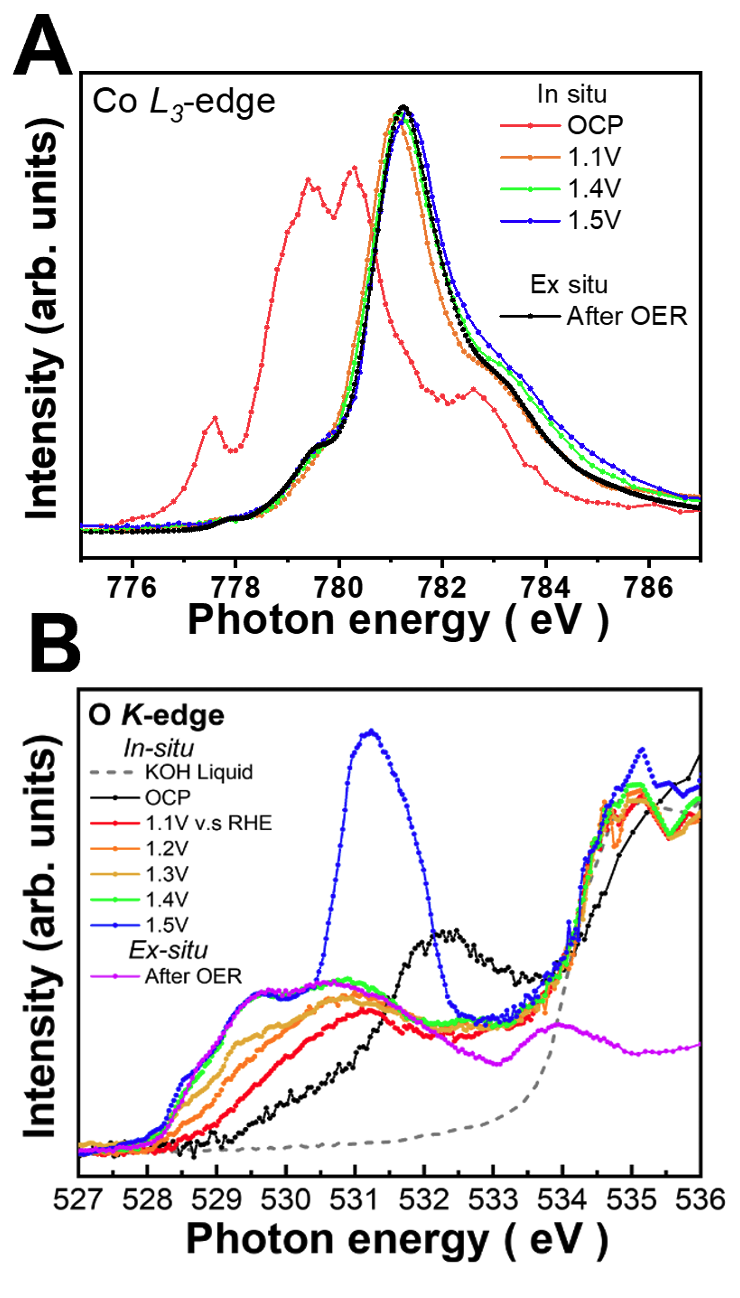


Figure S11. *Operando* sXAS characterization of high-valent Co as an active site during OER. **(A)** Co *L_3_*-edge and **(B)** O *K*-edge of CoFe-[Cr(C_2_O_4_)_3_]^3-^-LDHs and ex-situ spectra of CoFe-[Cr(C_2_O_4_)_3_]^3-^-LDHs after OER for comparison (black circles, in vacuum).


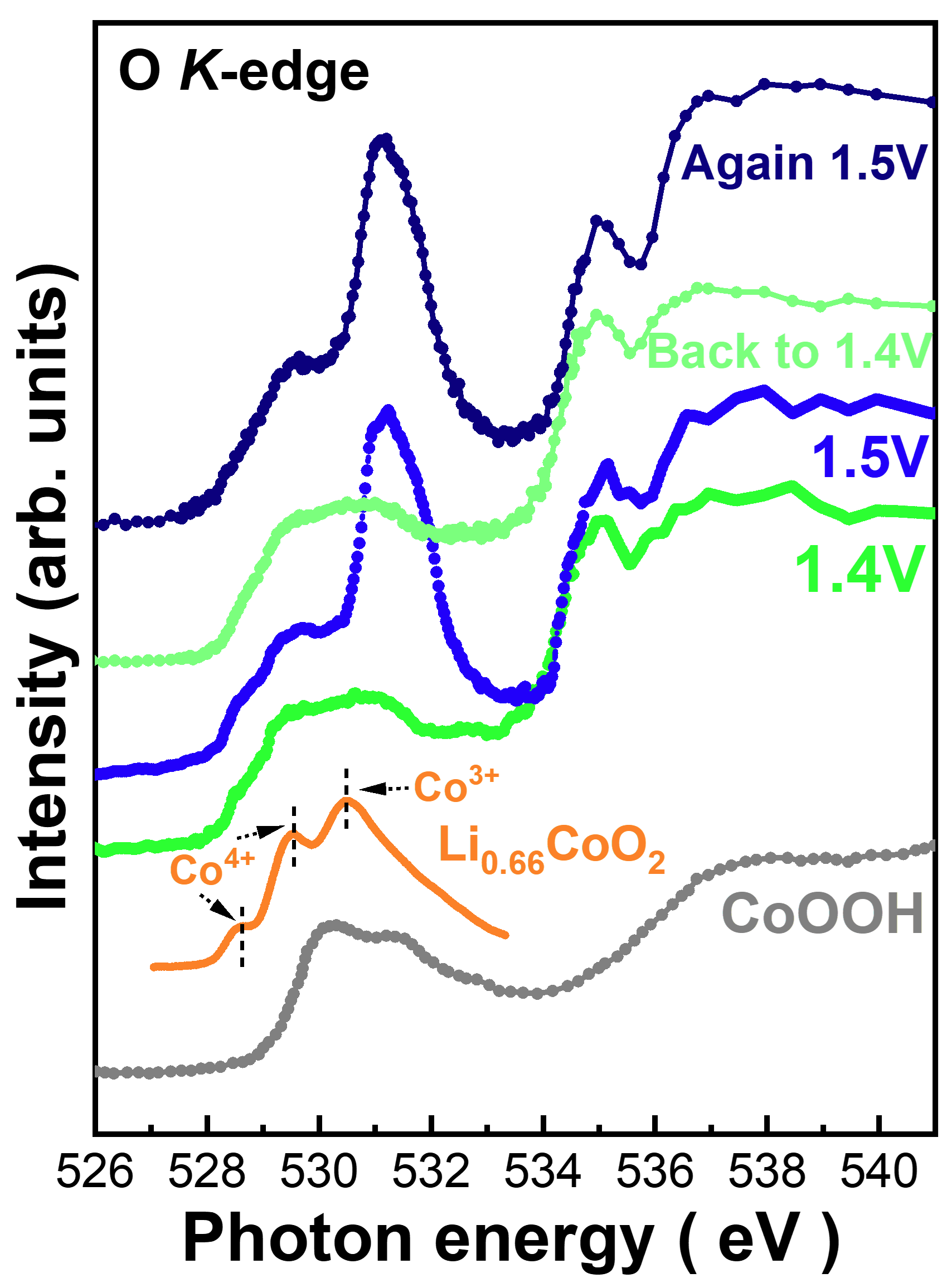


Figure S12. *Operando* sXAS reversible test of CoFe-[Cr(C_2_O_4_)_3_]^3-^-LDHs at O *K*-edge. Also included are the spectra of mixed-valent Co oxide Li_0.66_CoO_2_ (green line)^[3]^ and reference CoOOH.


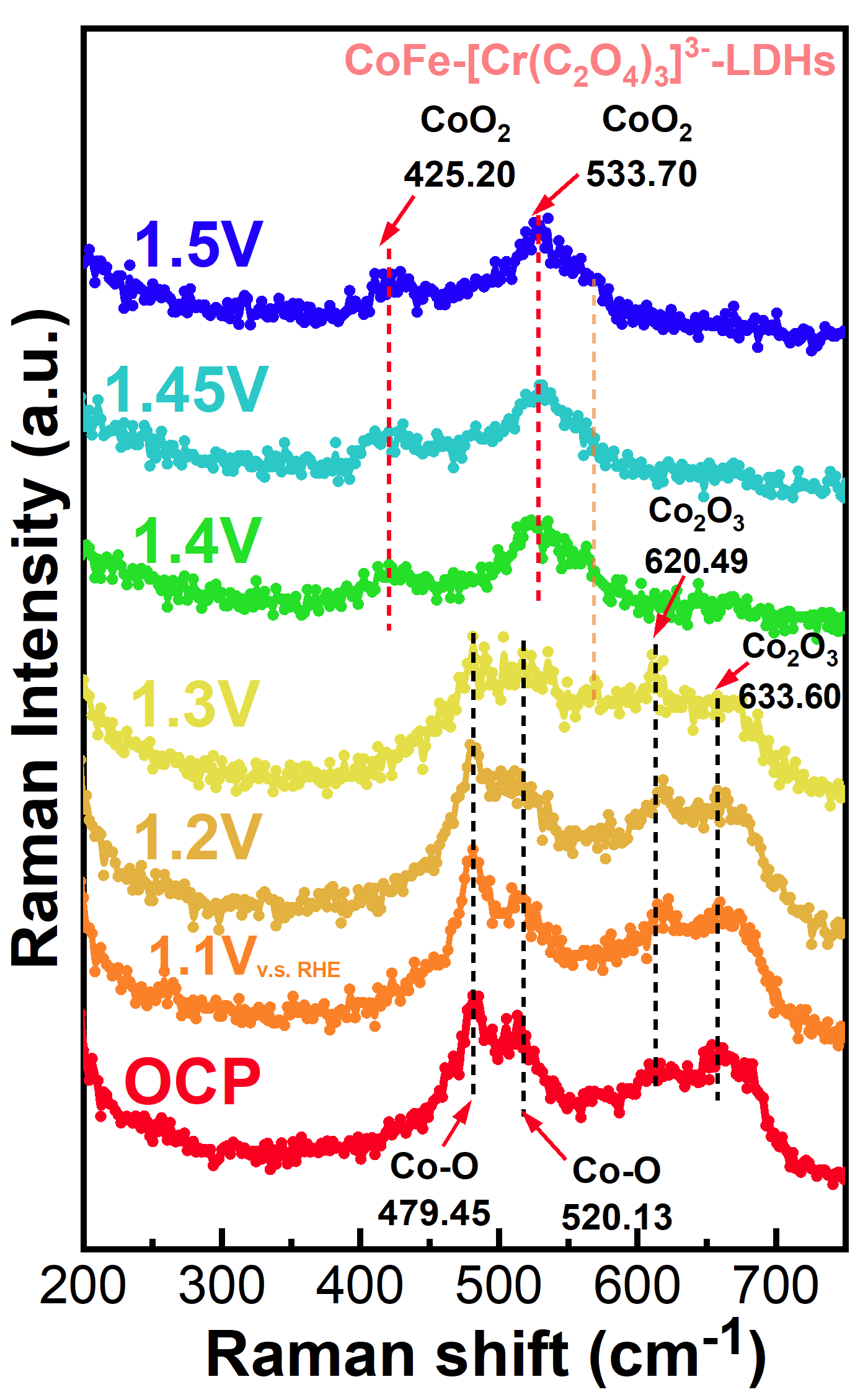


Figure S13. *In-situ/operando* Raman of CoFe-[Cr(C_2_O_4_)_3_]^3-^-LDHs.

Figure S14. Raman spectra for selected oxygen concentrations for the 18 O samples and for the high-valence (1.5V vs RHE) Co site during OER.

The in-situ Raman spectroscopy in 1 M KO(18)H electrolyte is carried out. As shown in Figure S14, the peak at 790 and 1554 cm^-1^ shift to lower wavenumber of 746 m^-1^ and 1468 cm^-1^ corresponding to (O_2_)^-n^ and O-O bonds, respectively according to Eq.1.

$\gamma=\frac{\vartheta(O18O18)}{\vartheta(O16O16)}=\frac{\sqrt{m\left( O18 \right)+m(O18)}}{\sqrt{m\left( O18 \right)*m(O18)}}/\frac{\sqrt{m\left( O16 \right)+m(O16)}}{\sqrt{m\left( O16 \right)*m(O16)}}$ Eq.1.

Experimental detail: H_2_O(18)(98 atom % O18) was purchased from Adamas-beta.

Figure S15. *Operando* sXAS of CoFe-[Cr(C_2_O_4_)_3_]^3-^-LDHs at O *K*-edge.


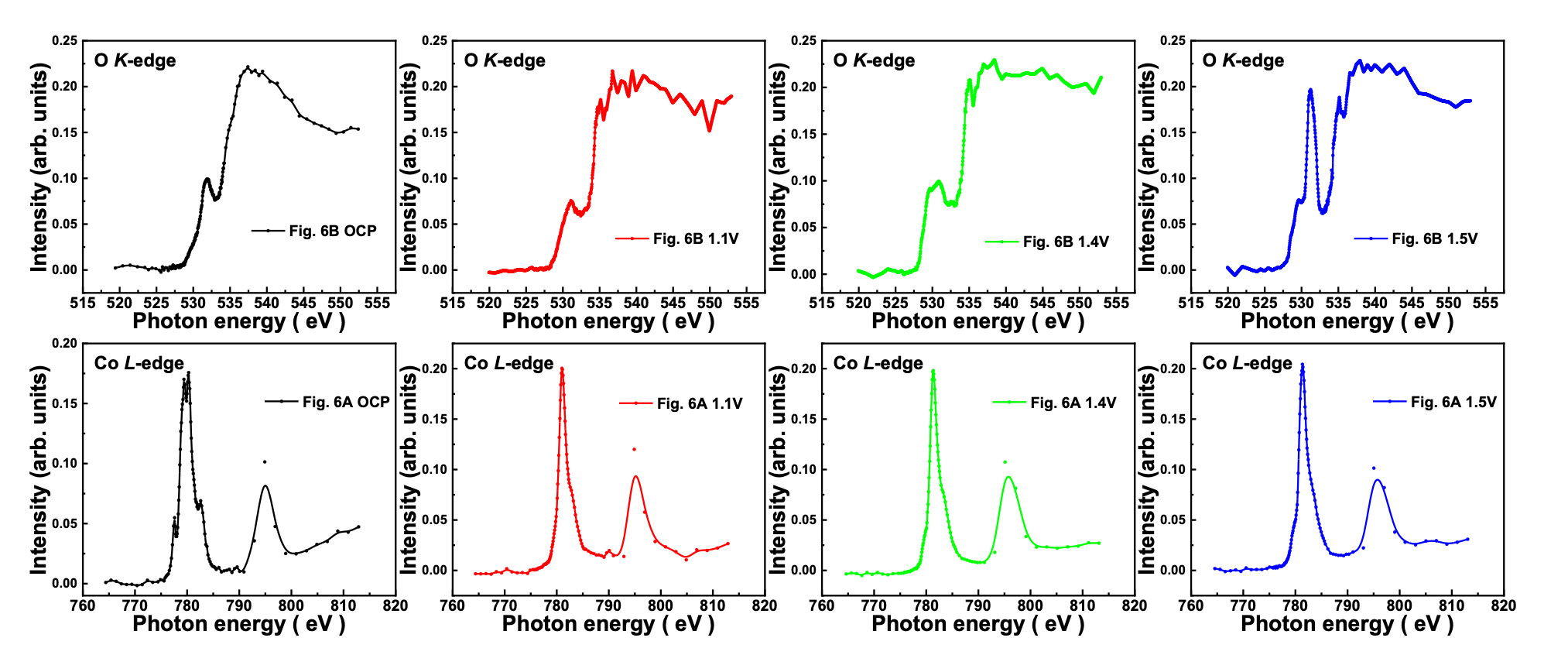


Figure S16. All raw sXAS operando data prior to background subtraction or data handling.

Figure S17. Soft X-ray in-situ testing with a loading of one-third of originally used loading.

A loading of one third that used originally has been conducted. As shown in Figure S17, the Co L_3_-edge signal is much weaker than the spectrum in air (black line) after the electrolyte is pumped into the cell (red line), but it remains visible. However, the pre-edge spectral features at the O K-edge become invisible, being significantly influenced by the electrolyte.

Table S1. Comparison of the OER activity of CoFe-[Cr(C_2_O_4_)_3_]^3-^-LDHs with other Co-Fe based bimetallic electrocatalysts in alkaline electrolyte.

| Catalysts | Electroyte | Overpotential at 10 mVcm^-2^ (mV) | Substrate | Reference |
| --- | --- | --- | --- | --- |
| CoFe-[Cr(C_2_O_4_)_3_]^3-^-LDHs | **1M KOH** | **248mV** | **GC** | **This work** |
| Na_2_Co^2+^[Fe^2+^(CN)_6_]·nH_2_O | 1M KOH | 255mV | GC | *ACS Catal*., **2022**, 12, 3138−3148 |
| CoFe-MOF-OH | 1M KOH | 265mV | GC | *ACS Catal*., **2019**, 9, 7356-7364 |
| CoFe-LDH | 1M KOH | 275mV | GC | *Chem. Eur. J.,* **2018**, 24, 4724-4728 |
| CoFe LDHs nanosheets | 1M KOH | 321mV | GC | *Angew. Chem. Int. Ed.*, **2017**, 56, 5867. |
| Fe-CoOOH/G | 1M KOH | 330mV | GC | *Adv. Energy. Mater.*, **2017**, 7, 1602148 |
| NaCo_0.8_Fe_0.2_O_2_ | 1M KOH | 330mV | GC | *Energ. Environ. Sci.*, **2017**, 10, 121-128. |

■ **References**

[1] C.-W. Tung, Y.-Y. Hsu, Y.-P. Shen, Y. Zheng, T.-S. Chan, H.-S. Sheu, Y.-C. Cheng, H. M. Chen, *Nature communications* **2015**, 6, 8106.

[2] Peng Jiang,Jeng-Lung Chen,[Ferenc Borondics](https://www.sciencedirect.com/author/6506278300/ferenc-borondics),Per-Anders Glans,Mark W. West,Ching-Lin Chang,[Miquel Salmeron](https://www.sciencedirect.com/author/24318371900/miquel-b-salmeron), Jinghua Guo, *Electrochemistry Communications*, **2010**, 12(6), 820-822.

[3] T. Mizokawa, Y. Wakisaka, T. Sudayama, C. Iwai, K. Miyoshi, J. Takeuchi, H. Wadati, D. Hawthorn, T. Regier, G. Sawatzky, *Physical review letters* **2013**, 111, 056404.
